# Assessment of Mortality Among Durable Left Ventricular Assist Device Recipients Ineligible for Clinical Trials

**DOI:** 10.1001/jamanetworkopen.2020.32865

**Published:** 2021-01-08

**Authors:** Alexander A. Brescia, Tessa M. F. Watt, Francis D. Pagani, Thomas M. Cascino, Min Zhang, Jeffrey S. McCullough, Supriya Shore, Donald S. Likosky, Keith D. Aaronson, Michael P. Thompson

**Affiliations:** 1 Department of Cardiac Surgery, Michigan Medicine, University of Michigan, Ann Arbor; 2 Center for Healthcare Outcomes and Policy, University of Michigan, Ann Arbor; 3 Division of Cardiovascular Medicine, Department of Internal Medicine, Michigan Medicine, University of Michigan, Ann Arbor; 4 Department of Biostatistics, School of Public Health, University of Michigan, Ann Arbor; 5 Department of Health Management and Policy, School of Public Health, University of Michigan, Ann Arbor

## Abstract

**Importance:**

While wide-scale adoption of durable left ventricular assist devices (LVADs) can be attributed to favorable randomized clinical trial outcomes, restrictive selection criteria may be associated with a lack of generalizability to real-world experience.

**Objective:**

To estimate the proportion of LVAD recipients who are eligible for clinical trials and to assess whether an association exists between trial eligibility and mortality.

**Design, Setting, and Participants:**

This cohort study examined 14 679 patients undergoing primary, intracorporeal continuous-flow LVAD implantation (with or without a right ventricular assist device) in 181 North American centers from January 1, 2012, to June 30, 2017, identified in the Interagency Registry for Mechanically Assisted Circulatory Support (INTERMACS). To simulate a trial population, trial criteria from the Multicenter Study of MagLev Technology in Patients Undergoing Mechanical Circulatory Therapy With HeartMate 3 (MOMENTUM 3) were mapped to INTERMACS variables. Patients were categorized as eligible for trial inclusion or ineligible for trial inclusion and by number of ineligibility criteria met. Follow-up in INTERMACS was complete for all patients through October 31, 2017. Data were analyzed from July 2019 through November 2020.

**Exposures:**

Undergoing durable LVAD implantation.

**Main Outcomes and Measures:**

Trial eligibility and postimplant mortality were analyzed using Kaplan-Meier estimates and Cox proportional hazards models.

**Results:**

Among 14 679 recipients, mean (SD) age was 57 (13) years, 11 503 individuals (78.4%) were men, and 11 406 individuals (77.7%) presented with New York Heart Association class IV heart failure. A total of 6429 recipients (43.8%) were ineligible for trial inclusion, of whom 4226 individuals (65.7%) met 1 ineligibility criterion, 1442 individuals (22.4%) met 2 criteria, and 761 individuals (11.8%) met 3 or more criteria. Estimated mortality for recipients who were trial-ineligible was higher than for recipients who were trial-eligible (1-year mortality: 25.3% [95% CI, 24.2%-26.5%] vs 16.2% [95% CI, 15.4%-17.1%]; 3-year mortality: 42.8% [95% CI, 41.3%-44.4%] vs 36.4% [95% CI, 35.0%-37.8%]; log-rank *P* < .001 for both). Patients who were trial-ineligible had increased risk of mortality compared with patients who were trial-eligible if they met 1 trial ineligibility criterion (hazard ratio [HR], 1.16 [95% CI, 1.08-1.24]; *P* < .001), 2 trial ineligibility criteria (HR, 1.51 [95% CI, 1.36-1.67]; *P* < .001), or 3 or more trial ineligibility criteria (HR, 2.09 [95% CI, 1.84-2.39]; *P* < .001). Among patients meeting only 1 ineligibility criterion, 4 criteria were independently associated with mortality: prior or ongoing mechanical circulatory support (HR, 1.63 [95% CI, 1.23-2.16]; *P* = .001), elevated creatinine level (HR, 1.42 [95% CI, 1.17-1.72]; *P* < .001), elevated bilirubin level (HR, 1.39 [95% CI, 1.17-1.66]; *P* < .001), and low albumin or prealbumin level (HR, 1.18 [95% CI, 1.05-1.33]; *P* = .007).

**Conclusions and Relevance:**

These findings suggest that while treatment for patients who are ineligible for LVAD trial inclusion should be weighed against medical management, more consideration could be given to designing trials with eligibility criteria that reflect real-world experience.

## Key Points

**Question:**

What proportion of real-world recipients of left ventricular assist devices (LVADs) meet clinical trial eligibility, and what prognoses are associated with ineligibility for randomized clinical trials?

**Findings:**

In this cohort study of 14 679 real-world recipients of durable LVADs, a total of 43.8% were ineligible for trial inclusion. Each additional criterion of ineligibility met by participants was associated with an increase in risk of mortality.

**Meaning:**

These findings suggest that while treatment for individuals ineligible for clinical trials should be weighed against medical management, more consideration could be given to designing trials with eligibility criteria that reflect real-world experience.

## Teaser

This cohort study estimates the proportion of recipients of durable left ventricular assist devices (LVADs) who are ineligible for clinical trial participation and assesses whether an association exists between ineligibility and mortality.

## Introduction

Durable left ventricular assist device (LVAD) implantation is an accepted surgical treatment for advanced heart failure refractory to guideline-directed medical therapy, with more than 25 000 implantations in the Interagency Registry for Mechanically Assisted Circulatory Support (INTERMACS) to date.^1,2^ The rise in use of durable LVAD has followed clinical trials^3,4,5,6,7,8,9^ of newer continuous-flow rotary pumps demonstrating improved outcomes with fewer adverse events. These clinical trial outcomes^4,5,6,7,8,9,10^ were followed by US Food and Drug Administration (FDA) approval for commercial use for both bridge-to-transplant and destination therapy indications, and this approval has broadened the application of LVAD therapy. To date, use of LVAD therapy in practice remains guided by clinical trial findings, which are derived from a highly selected pool of patients.

However, the extent to which recipients of durable LVADs in practice meet clinical trial eligibility criteria is unknown. If real-world patients receiving LVADs have different outcomes than patients in clinical trials, clinicians should understand these differences when informing patients about the expected benefits and prognosis of LVAD therapy. Applying trial eligibility criteria to real-world recipients of durable LVADs may help determine the impact of trial inclusion and exclusion criteria on mortality. This may help clinicians better understand whether clinical trial outcomes can be generalized to patients who would have been ineligible for the trials that led to the commercial availability of these devices.

The objectives of this study were to determine the generalizability of LVAD clinical trial findings by (1) using INTERMACS to characterize trial eligibility among real-world recipients of LVADs, (2) comparing mortality between recipients who were eligible for trial inclusion and those who were ineligible, and (3) examining associations of specific trial-ineligibility criteria with long-term mortality to inform patient prognosis. We hypothesized that LVAD recipients who did not meet trial criteria would have increased risk and worse risk-adjusted outcomes compared with recipients who met trial criteria.

## Methods

### Data Source

The University of Michigan Institutional Review Board approved this cohort study and waived the requirement for informed consent because it met the board’s 4 criteria for a waiver: (1) the research involved no more than minimal risk to participants; (2) research could not practicably (ie, feasibly) be carried out without the waiver or alteration; (3) the waiver or alteration did not adversely affect the rights or welfare of the study participants; and (4) whenever appropriate, the participants or their legally authorized representative were provided with additional pertinent information after participation. This study followed the Strengthening the Reporting of Observational Studies in Epidemiology (STROBE) reporting guideline. The primary data source was INTERMACS, previously funded in part by the National Heart, Lung, and Blood Institute (NHLBI) under contract No. HHSN268201100025C and a joint effort among the NHLBI, FDA, and Centers for Medicare & Medicaid Services (CMS). The registry was established in 2005 at the University of Alabama at Birmingham and is currently administered by The Society of Thoracic Surgeons. This study was undertaken prior to the acquisition of INTERMACS by The Society of Thoracic Surgeons. The registry is prospectively maintained and includes a real-world population of patients undergoing FDA-approved implantation of durable mechanical circulatory support (MCS) devices, including LVADs, right ventricular assist devices, and total artificial hearts. The study period coincided with 3 trials: ENDURANCE,^4^ ENDURANCE supplement,^5^ and the Multicenter Study of MagLev Technology in Patients Undergoing Mechanical Circulatory Therapy with HeartMate 3 (HM3) (MOMENTUM 3).^6,7,8,9^ Some HeartMate II (HMII; Abbott Laboratories) devices in INTERMACS may have been in MOMENTUM 3 as control devices, but MOMENTUM 3 study devices (ie, HM3s; Abbott Laboratories) were not included in INTERMACS. Although INTERMACS has a variable for study inclusion, the variable is frequently missing and unverifiable, so we were unable to determine which patients were in a clinical trial.

The data set for this study was provided by the INTERMACS Data and Clinical Coordinating Center to the University of Michigan through permission from the NHLBI. The study was performed under a data use agreement with the Research Data Assistance Center.

### Patient Population

Patients undergoing primary, intracorporeal continuous-flow LVAD implantation (with or without right ventricular assist device) between January 1, 2012, and June 30, 2017, were identified in INTERMACS. Patients receiving an isolated right ventricular assist device or total artificial heart and those with a history of durable MCS device implantation when entered into INTERMACS were excluded. Each patient was categorized as eligible for trial inclusion or ineligible for trial inclusion through 2 analyses, using inclusion and exclusion criteria from MOMENTUM 3 applied to INTERMACS variables (eTable 1 in the Supplement). Eligibility criteria in MOMENTUM 3 included 7 inclusion and 25 exclusion criteria.^6^

The primary (ie, limited) analysis included defined trial inclusion and exclusion criteria that could be directly mapped to INTERMACS (eg, bilirubin >2.5 mg/dL [to convert to micromoles per liter, multiply by 17.104] and creatinine >2.5 mg/dL [to convert to micromoles per liter, multiply by 88.4]) (eTable 2 in the Supplement). We excluded 2 inclusion criteria that were not feasible to match to INTERMACS variables: signing an informed consent form and female patients of childbearing age agreeing to use adequate contraception. The primary analysis captured all 5 remaining MOMENTUM 3 inclusion criteria and 11 of 25 exclusion criteria.

To use INTERMACS variables to identify more patients who would have been eligible for the MOMENTUM 3 trial, an additional analysis (ie, comprehensive) was performed matching MOMENTUM and INTERMACS variables that were similar but not identical (eg, MOMENTUM exclusion variable: presence of an active, uncontrolled infection; INTERMACS exclusion variable: events this hospitalization — major infection [yes]) (eTable 1 in the Supplement). The comprehensive analysis captured the same 5 inclusion criteria as the primary analysis and 21 of 25 exclusion criteria.

### Outcomes

The primary outcome was trial eligibility. Patients failing to meet 1 or more inclusion criteria or meeting 1 or more exclusion criteria were considered trial-ineligible, while patients meeting all inclusion criteria and no exclusion criteria were considered trial-eligible. Number of reasons for trial ineligibility was determined for each patient, and patients were categorized by number of ineligibility criteria they met (ie, 0, 1, 2, or ≥3).

The secondary outcome was postimplant mortality. Time-to-event analyses specified death as a failure event, while patients who underwent heart transplantation, explant without exchange, or LVAD decommissioning with no new device or had other reason for LVAD removal were censored. Patients undergoing LVAD exchange remained in the analysis and were not censored. Death status and event dates were collected from INTERMACS, with complete follow-up performed for all patients through October 31, 2017.

### Statistical Analysis

Bivariate comparisons used 2-sample *t *tests or χ^2^ tests, as appropriate. Missing preoperative variables were treated as a separate category. Missing values for trial exclusion criteria were considered not present, which provided the most conservative estimates of trial ineligibility.

Time-to-event analyses were performed using log-rank test and Kaplan-Meier estimates to compare cumulative mortality by trial eligibility status (ineligible vs eligible), number of trial ineligibility criteria per patient (ie, 0, 1, 2, or ≥3), INTERMACS patient profile of advanced heart failure number (ie, 1, 2, 3, or 4-7),^11^ and trial eligibility status stratified by INTERMACS profile. Sensitivity analyses included comparing mortality by eligibility status after stratifying by device type and treating transplantation as a competing risk to mortality rather than a censored event.

Multivariable Cox models incorporating clinical characteristics and device type were created to report risk-adjusted mortality with hazard ratios (HRs) and 95% CIs; specific hospital for procedure was included as a random effect through a shared frailty model. Cox models were created to compare patients ineligible for trial vs those eligible for trial (eTable 3 in the Supplement), then to compare patients meeting 1, 2, or 3 or more trial ineligibility criteria. The number of trial ineligibility criteria met (ie, 0, 1, 2, or ≥3) was considered as both a categorical and continuous variable in separate analyses. Lastly, the most frequent reasons for exclusion were evaluated for association with mortality through Cox models in 3 ways: with any number of exclusions present, when each exclusion was the only criterion present, and when the specific criterion plus 1 or more criteria were present. All models were adjusted for age, sex, body mass index (BMI, calculated as weight in kilograms divided by height in meters squared), blood type O, White race, implantable cardioverter defibrillator presence, INTERMACS patient profile, bridge-to-transplant status, prior coronary artery bypass or valve surgery, concomitant cardiac surgery, device type, and year of implant.

Statistical significance was defined as *P* < .05, and *P* values were 2-sided. All analyses were performed in Stata statistical software version 16.0 (StataCorp) from July 2019 to November 2020.

## Results

### Patient Population and Trial Eligibility

Among 14 679 recipients of LVAD, mean (SD) age was 57 (13) years, 11 503 (78.4%) individuals were men, 9704 individuals (66.1%) were White, mean (SD) BMI was 28.6 (7.1), and 11 406 individuals (77.7%) presented with New York Heart Association class IV heart failure (Table 1). In total, 2489 patients (17.0%) were INTERMACS patient profile 1, 5041 patients (34.3%) were profile 2, 4879 patients (33.2%) were profile 3, and 2186 patients (14.9%) were profiles 4 through 7. The HMII was implanted in 10 652 patients (72.6%), and HeartWare LVAD (Medtronic) was implanted in 4027 patients (27.4%).

**Table 1.** Patient Characteristics by Trial Eligibility Status

Characteristic	Patients, No. (%)			*P* value
	Overall (N = 14 679)	Trial eligible (n = 8250)	Trial ineligible (n = 6429)	
Age, mean (SD), y	56.9 (13.0)	56.9 (12.9)	56.9 (13.1)	.96
Sex				
Men	11 503 (78.4)	6421 (77.8)	5082 (79.0)	.24
Women	3153 (21.5)	1815 (22.0)	1338 (20.8)	
Unknown	23 (0.2)	14 (0.2)	9 (0.1)	
White	9704 (66.1)	5401 (65.5)	4303 (66.9)	.06
Hispanic				
Yes	969 (6.6)	517 (6.3)	452 (7.0)	.03
No	13 469 (91.8)	7612 (92.3)	5857 (91.1)	
Unknown	241 (1.6)	121 (1.5)	120 (1.9)	
BMI, mean (SD)	28.6 (7.1)	28.9 (7.2)	28.2 (7.0)	<.001
Body surface area, mean (SD), m^2^	2.08 (0.31)	2.09 (0.31)	2.06 (0.32)	<.001
Blood type O	6822 (46.5)	3876 (47.0)	2946 (45.8)	.38
INTERMACS patient profile				
1	2489 (17.0)	676 (8.2)	1813 (28.2)	<.001
2	5041 (34.3)	2896 (35.1)	2145 (33.4)	
3	4879 (33.2)	3419 (41.4)	1460 (22.7)	
4-7	2186 (14.9)	1239 (15.0)	947 (14.7)	
Missing	84 (0.6)	20 (0.2)	64 (1.0)	
NYHA heart failure class				
I-II	165 (1.1)	0	165 (2.6)	<.001
III	2422 (16.5)	1524 (18.5)	898 (14.0)	
IV	11 406 (77.7)	6392 (77.5)	5014 (78.0)	
Missing	686 (4.7)	334 (4.0)	352 (5.5)	
LVEF, %				
≥50	24 (0.2)	0	24 (0.4)	<.001
40-49	69 (0.5)	0	69 (1.1)	
30-39	528 (3.6)	0	528 (8.2)	
20-29	3511 (23.9)	2117 (25.7)	1394 (21.7)	
<20	9766 (66.5)	5688 (68.9)	4078 (63.4)	
Not documented	728 (5.0)	422 (5.1)	306 (4.8)	
Unknown	53 (0.4)	23 (0.3)	30 (0.5)	
Bridge to transplant				
Listed	3704 (25.2)	2209 (26.8)	1495 (23.3)	<.001
Likely	2322 (15.8)	1310 (15.9)	1012 (15.7)	.82
Implantable cardioverter defibrillator	11 547 (78.7)	6873 (83.3)	4674 (72.7)	<.001
Concomitant cardiac surgery	6275 (42.7)	2908 (35.2)	3367 (52.4)	<.001
Prior coronary artery bypass grafting	2931 (20.0)	1642 (19.9)	1289 (20.0)	.83
Prior valve surgery	1023 (7.0)	539 (6.5)	484 (7.5)	.02
Inotrope	12 075 (82.3)	7056 (85.5)	5019 (78.1)	<.001
Device type				
HeartMate II	10 652 (72.6)	5977 (72.4)	4675 (72.7)	.72
HeartWare LVAD	4027 (27.4)	2273 (27.6)	1754 (27.3)	
Implant year				
2012	1591 (10.8)	867 (10.5)	724 (11.3)	.17
2013	2753 (18.8)	1575 (19.1)	1178 (18.3)	
2014	2886 (19.7)	1614 (19.6)	1272 (19.8)	
2015	3202 (21.8)	1846 (22.4)	1356 (21.1)	
2016	2878 (19.6)	1602 (19.4)	1276 (19.8)	
2017	1369 (9.3)	746 (9.0)	623 (9.7)	
Deaths	4287 (29.2)	2163 (26.2)	2124 (33.0)	<.001
Heart rate, mean (SD), beats per minute	89.3 (17.6)	88.6 (17.1)	90.2 (18.2)	<.001
Blood pressure, mean (SD), mm Hg				
Systolic	105.5 (16.1)	106.6 (15.5)	104.0 (16.8)	<.001
Diastolic	65.3 (11.5)	65.8 (11.1)	64.7 (11.9)	<.001
Laboratory values, mean (SD)				
Serum creatinine, mg/dL	1.39 (0.69)	1.30 (0.41)	1.50 (0.91)	<.001
Blood urea nitrogen, mg/dL	29.10 (18.05)	27.13 (15.06)	31.63 (21.02)	<.001
Serum sodium, mEq/L	135.07 (4.81)	135.12 (4.38)	135.01 (5.31)	.16
Hemoglobin, g/dL	11.18 (2.17)	11.62 (2.03)	10.61 (2.22)	<.001
Platelet count, No. × 10^3^/μL	195.47 (79.97)	209.58 (71.64)	177.20 (86.27)	<.001
Albumin, g/dL	3.40 (0.64)	3.59 (0.52)	3.16 (0.71)	<.001
Total bilirubin, mg/dL	1.40 (1.99)	1.01 (0.53)	1.90 (2.85)	<.001
Alanine aminotransferase, U/L^a^	67.25 (224.38)	47.37 (119.56)	92.37 (307.89)	<.001
Aspartate aminotransferase, U/L^a^	56.95 (218.24)	39.43 (95.38)	79.11 (308.98)	<.001
INR	1.31 (0.39)	1.22 (0.20)	1.41 (0.52)	<.001

Abbreviations: BMI, body mass index (calculated as weight in kilograms divided by height in meters squared); INR, international normalized ratio; INTERMACS, Interagency Registry for Mechanically Assisted Circulatory Support; LVAD, left ventricular assist device; LVEF, left ventricular ejection fraction; NYHA, New York Heart Association.

SI conversion factors: To convert alanine aminotransferase and aspartate aminotransferase to microkatals per liter, multiply by 0.0167; albumin to grams per liter, multiply by 10; blood urea nitrogen to micromoles per liter, multiply by 0.357; hemoglobin to grams per liter, multiply by 10.0; platelet count to × 10^9^ per liter, multiply by 1.0; serum creatinine to micromoles per liter, multiply by 88.4; serum sodium to micromoles per liter, multiply by 1.0; and total bilirubin to micromoles per liter, multiply by 17.104.

In total, 6429 patients (43.8%) were trial-ineligible in the limited analysis and 7888 patients (53.7%) were trial-ineligible in the comprehensive analysis (eFigure 1 in the Supplement). The percentage of patients who were ineligible for trial inclusion was consistent over the study period in these analyses (eFigure 2 in the Supplement). In the limited analysis, 8250 patients (56.2%) met no criteria conferring ineligibility (eg, were trial-eligible), 4226 patients (28.8%) met 1 ineligibility criterion, 1442 patients (9.8%) met 2 ineligibility criteria, and 761 patients (5.2%) met 3 or more ineligibility criteria (eFigure 3 in the Supplement). In the comprehensive analysis, 6791 patients (46.3%) met no ineligibility criteria, 4563 patients (31.1%) met 1 ineligibility criterion, 2015 patients (13.7%) met 2 ineligibility criteria, and 1310 patients (8.9%) met 3 or more ineligibility criteria (eFigure 3 in the Supplement). The most common reasons for trial ineligibility in both analyses were low albumin or prealbumin levels (2281 patients [15.5%]), thrombocytopenia (1369 patients [9.3%]), and elevated bilirubin levels (1293 patients [8.8%]) (eFigure 4 in the Supplement).

A greater proportion of patients who were trial-ineligible, compared with patients who were trial-eligible, had INTERMACS profile 1 (1813 patients [28.2%] vs 676 patients [8.2%]; *P* < .001) and underwent other concomitant cardiac surgery (3367 patients [52.4%] vs 2908 patients [35.2%]; *P* < .001). A greater proportion of patients who were trial-ineligible , compared with patients who were trial-eligible, had a left ventricular ejection fraction of less than 20% (4078 patients [63.4%] vs 5688 patients [68.9%]; *P* < .001), and a smaller proportion of patients had an implantable cardioverter defibrillator (4674 patients [72.7%] vs 6873 patients [83.3%]; *P* < .001) (Table 1).

### Postimplant Mortality

**Figure 1.**
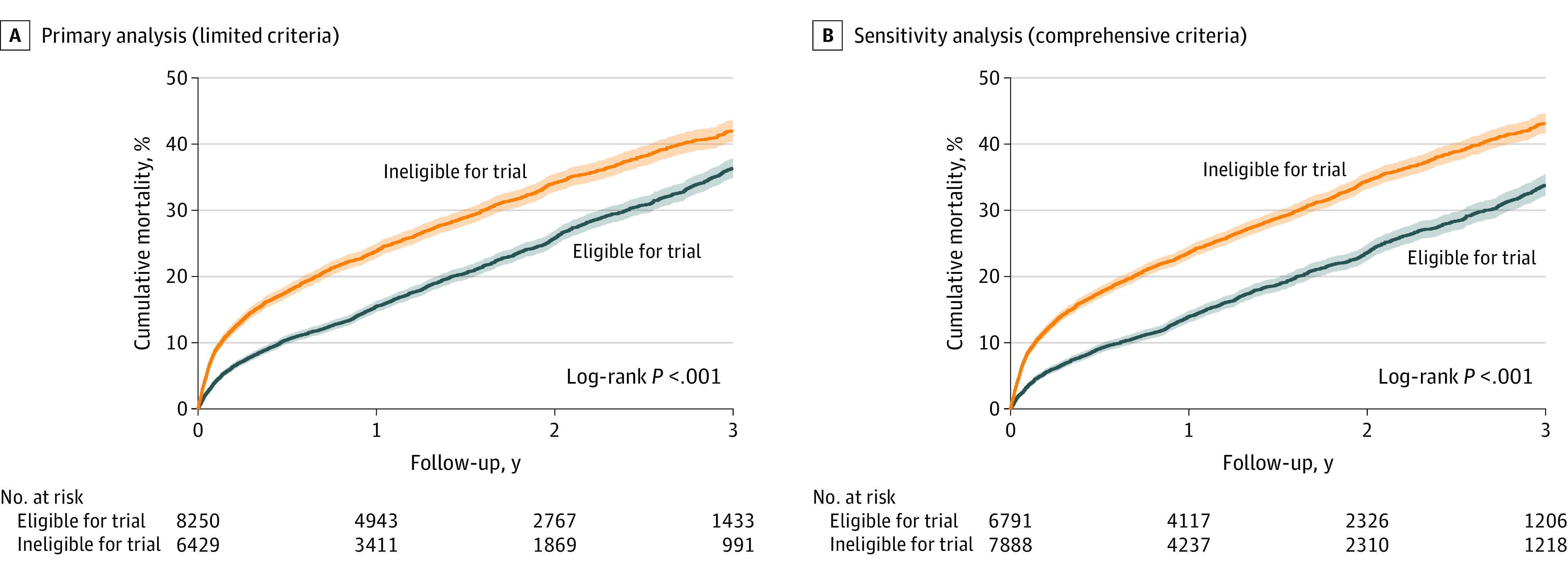
Kaplan-Meier Estimated Mortality by Trial Eligibility Status Shaded areas indicate 95% CIs.

In the limited analysis, 1-year mortality was higher for patients who were trial-ineligible compared with those who were trial-eligible (25.3% [95% CI, 24.2%-26.5%] vs. 16.2% [95% CI, 15.4%-17.1%]; *P* < .001) (Figure 1A). Similarly for the comprehensive analysis, 1-year mortality was higher for patients who were trial-ineligible compared with those who were trial-eligible (25.1% [95% CI, 24.1%-26.1%] vs. 14.5% [95% CI, 13.6%-15.5%]; *P* < .001) (Figure 1B). Additionally, 3-year mortality was higher for patients who were trial-ineligible compared with those who were trial-eligible in the limited analysis (42.8% [95% CI, 41.3%-44.4%] vs 36.4% [95% CI, 35.0%-37.8%]; log-rank *P* < .001) and the comprehensive analysis (43.8% [95% CI, 42.3%-45.2%] vs 33.9% [95% CI, 32.3%-35.4%]; log-rank *P* < .001). With cardiac transplantation as a competing risk, the cumulative incidence of mortality was higher for patients who were trial-ineligible compared with those who were trial-eligible in both analyses (eFigure 5 in the Supplement). These mortality differences were comparable between HMII and HeartWare VAD devices (eFigure 6 in the Supplement). Median (interquartile interval) follow-up was 1.2 (0.5-2.4) years, and this was longer for patients who were trial-eligible compared with those who were trial-ineligible (1.3 [0.6-2.5] years vs 1.1 [0.5-2.3] years; *P* < .001). Trial ineligibility was independently associated with increased risk of mortality in the limited analysis (HR, 1.29 [95% CI, 1.21-1.37]; *P* < .001) and comprehensive analysis (HR, 1.42 [95% CI, 1.33-1.52]; *P* < .001) (Table 2; eTable 3 in the Supplement).

**Table 2.** Cox Proportional Hazards Model for Overall Mortality by Trial Eligibility Status and Number of Exclusions Met

Eligibility status	Limited analysis		Comprehensive analysis	
	HR^a^ (95% CI)	*P* value	HR^a^ (95% CI)	*P* value
Trial eligible	1 [Reference]	NA	1 [Reference]	NA
Trial ineligible	1.29 (1.21-1.37)	<.001	1.42 (1.33-1.52)	<.001
Criteria met for ineligibility, No.				
0	1 [Reference]	NA	1 [Reference]	NA
1	1.16 (1.08-1.24)	<.001	1.29 (1.19-1.38)	<.001
2	1.51 (1.36-1.67)	<.001	1.54 (1.40-1.69)	<.001
≥3	2.09 (1.84-2.39)	<.001	2.10 (1.88-2.34)	<.001
Risk per each additional ineligibility criterion met	1.22 (1.18-1.27)	<.001	1.22 (1.19-1.25)	<.001

Abbreviations: HR, hazard ratio; NA, not applicable.

^a^ The reference group for all Cox HRs was patients who were trial-eligible.

**Figure 2.**
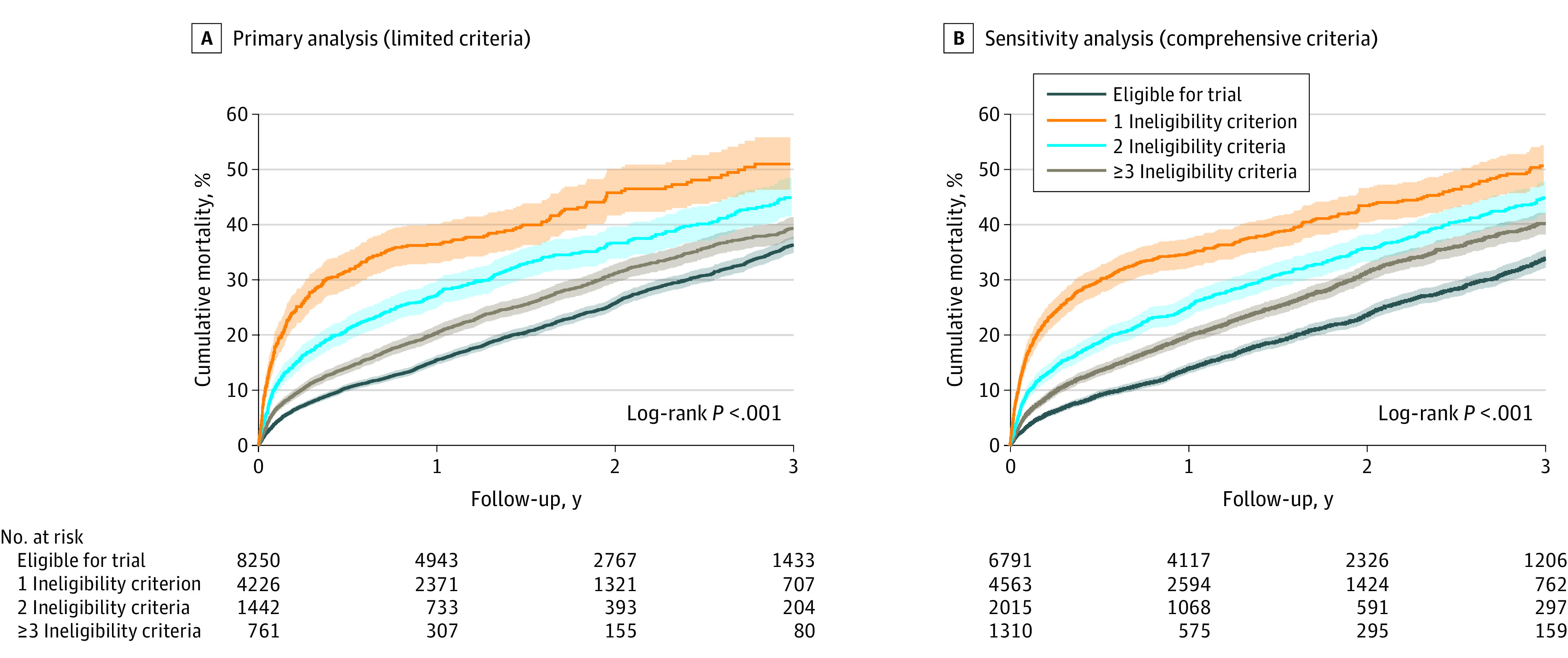
Kaplan-Meier Estimated Mortality by Number of Ineligibility Criteria Shaded areas indicate 95% CIs.

Number of ineligibility criteria per patient was associated with mortality in both analyses, with higher estimated mortality for each additional criterion for trial ineligibility (Figure 2). Estimated mortality at 1 year for patients meeting 3 or more ineligibility criteria was 39.9% (95% CI, 36.3%-43.7%) in the limited analysis and 37.6% (95% CI, 34.9%-40.4%) in the comprehensive analysis; estimated 1-year mortality for patients who were trial-eligible was 16.2% [95% CI, 15.4%-17.1%] in the limited analysis and 14.5% [95% CI, 13.6%-15.5%] in the comprehensive analysis. The full distribution of criteria determining trial ineligibility is detailed in eTable 4 in the Supplement.

**Figure 3.**
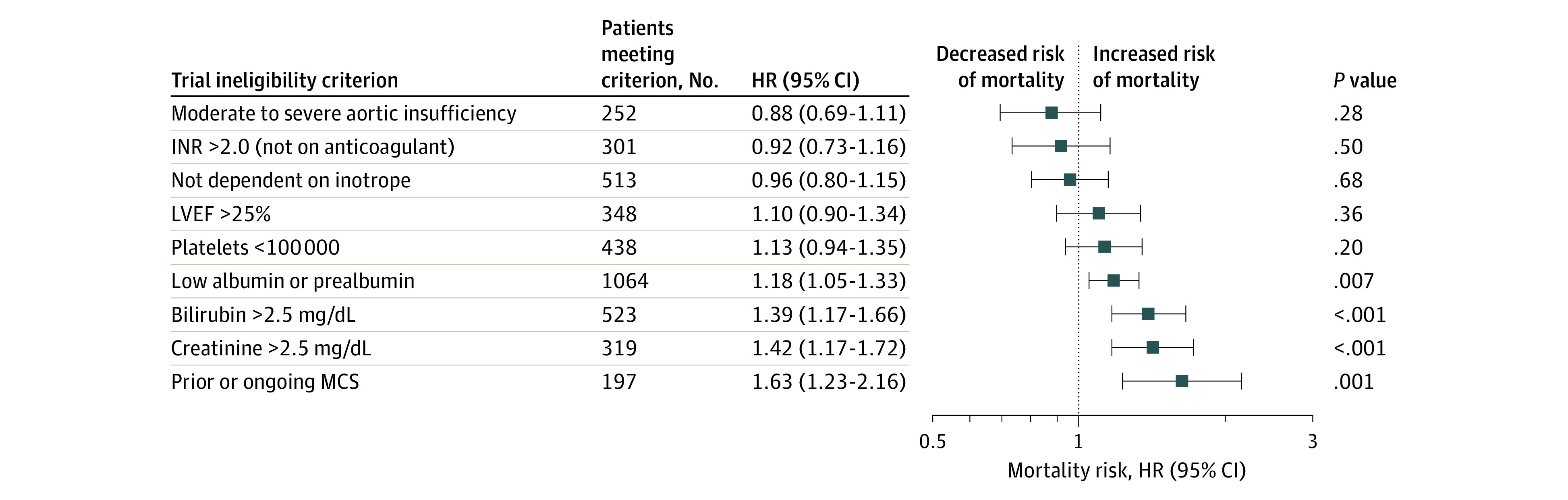
Risk of Mortality by Ineligibility Criteria Forest plot of specific trial criteria associated with increased risk of mortality according to multivariable Cox proportional hazards models for patients who were trial-eligible and met only 1 ineligibility criterion. HR indicates hazard ratio; INR, international normalized ratio; LVEF, left ventricular ejection fraction; MCS, mechanical circulatory support. To convert bilirubin to micromoles per liter, multiply by 17.104 and creatinine to micromoles per liter, multiply by 88.4.

An increased number of trial ineligibility criteria was associated with increased mortality risk for the limited analysis (1 criterion: HR, 1.16 [95% CI, 1.08-1.24]; *P* < .001; 2 criteria: HR, 1.51 [95% CI, 1.36-1.67]; *P* < .001; ≥3 criteria: HR, 2.09 [95% CI, 1.84-2.39]; *P* < .001) and comprehensive analysis (1 criterion: HR, 1.29 [95% CI, 1.19-1.38]; *P* < .001; 2 criteria: HR, 1.54 [95% CI, 1.40-1.69]; *P* < .001; ≥3 criteria: HR, 2.10; [95% CI, 1.88-2.34]; *P* < .001). Mean increased mortality risk per additional ineligibility criterion was similar in the 2 analyses (Table 2). Among patients meeting only 1 ineligibility criterion in the limited analysis, 4 ineligibility criteria were independently associated with mortality: prior or ongoing MCS other than intra-aortic balloon pump (HR, 1.63 [95% CI, 1.23-2.16]; *P* = .001), serum creatinine greater than 2.5 mg/dL (HR, 1.42 [95% CI, 1.17-1.72]; *P* < .001), bilirubin greater than 2.5 mg/dL (HR, 1.39 [95% CI, 1.17-1.66]; *P* < .001), and low albumin or prealbumin (HR, 1.18 [95% CI, 1.05-1.33]; P = .007); no independent association was found for meeting any other single criterion (Figure 3). Meeting any 2 or more ineligibility criteria was associated with an increased risk of mortality (eTable 5 in the Supplement). In addition, meeting 3 or more ineligibility criteria was associated with an even higher risk of mortality, with the specific combination of low albumin or prealbumin, elevated bilirubin, and elevated creatinine levels associated with the worst prognosis (eTable 6 in the Supplement).

Among INTERMACS profiles, 1-year mortality was highest for profile 1 (28.8% [95% CI, 26.9%-30.8%]; log-rank *P* < .001) compared with profile 2 (20.6% [95% CI, 19.4%-21.8%]), profile 3 (16.8% [95% CI, 15.7%-17.9%]), and profiles 4 through 7 (17.4% [95% CI, 15.8%-19.2%]), with no significant difference between profile 3 and profiles 4 through 7 (eFigure 7 in the Supplement). Stratified by profile, 1-year mortality in the limited analysis differed between patients who were trial-ineligible vs those who were trial-eligible among patients with profile 1 (33.0% [95% CI, 30.8%-35.4%] vs 17.2% [95% CI, 14.3%-20.5%]; *P* < .001), profile 2 (25.3% [95% CI, 23.4%-27.3%] vs 17.1% [95% CI, 15.7%-18.6%]; *P* < .001), and profile 3 (20.5% [95% CI, 18.4%-22.8%] vs 15.2% [95% CI, 14.0%-16.5%]; *P* < .001) (eFigure 8 in the Supplement). In contrast, mortality did not differ by trial eligibility status among patients with profiles 4 through 7 (18.5% [95% CI, 16.1%-21.3%] vs 16.6% [95% CI, 14.5%-18.9%]; *P* = .44) (eFigure 8 in the Supplement). In the comprehensive analysis, patients who were trial-ineligible in all 4 groups of profiles had higher mortality compared with patients who were trial-eligible (eFigure 9 in the Supplement).

## Discussion

This cohort study of nearly 15 000 patients receiving primary, durable LVAD implants in INTERMACS found that approximately one-half of patients were ineligible for clinical trial enrollment based on MOMENTUM 3 trial criteria. Ineligible status was associated with higher mortality compared with being eligible for trial inclusion, with mortality estimates of patients who were trial-eligible comparable to postimplant mortality rates published in recent clinical trials.^5,6,7,8^ Additionally, prior or ongoing MCS (other than intra-aortic balloon pump), elevated creatinine levels, elevated bilirubin levels, and low albumin or prealbumin levels were isolated ineligibility criteria independently associated with mortality. Meeting any other single ineligibility criterion was not associated with increased risk of mortality. In contrast, meeting any 2 or more ineligibility criteria was associated with increased risk of mortality compared with being eligible for trial inclusion, with each additional ineligibility criterion associated with increased mortality risk, independent of device type. Collectively, these data suggest that not meeting clinical trial eligibility criteria was associated with different mortality rates compared with being eligible for trial inclusion and that meeting increased numbers of ineligibility criteria was associated with progressively increased mortality.

Multiple landmark clinical trials^3,4,5,6,7,8,9^ have demonstrated improved outcomes and fewer adverse events with the use of newer axial and centrifugal, continuous-flow, durable LVAD devices for both bridge-to-transplant and destination therapy. Most recently, in the MOMENTUM 3 trial,^6,7,8^ a totally magnetically levitated centrifugal-flow LVAD (HM3) was demonstrated to be superior to an axial-flow LVAD (HMII), with 77% of patients free from death, disabling stroke, or reoperation at 2 years. While clinical trial outcomes have been promising, the generalizability of these findings to the current real-world LVAD population has remained unclear. By evaluating generalizability within a contemporaneous real-world cohort, this study’s data may not only inform patient prognosis but may also be used for future clinical trial design, treatment decision-making, and informing regulatory policy for LVAD use and reimbursement.

Importantly, in addition to a higher risk of mortality for patients who were trial-ineligible, the number of ineligibility criteria (ie, meeting 1, 2, or 3 or more criteria) and the specific types of ineligibility criteria varied in their association with mortality. The expected findings that temporary MCS other than intra-aortic balloon pump, elevated bilirubin or creatinine levels,^12^ and low albumin or prealbumin levels in isolation each conferred worse mortality in patients who were otherwise-eligible for trial inclusion should be used by clinicians implanting commercial LVADs to improve prognostication and facilitate shared decision-making. Despite the knowledge that ineligibility factors, such as low albumin or prealbumin and elevated bilirubin levels, are associated with mortality and morbidity, the prevalence of trial ineligibility among recipients of LVADs has not changed over time.^13^ The FDA and clinical trialists may use findings from this study to refine future trial eligibility criteria to be more representative of real-world LVAD recipients and may consider eliminating criteria that may have no association with mortality (eg, thrombocytopenia). Understanding the association of each eligibility criterion with mortality may allow clinicians to better prognosticate, individualize treatment plans, and counsel candidates for LVAD on risks and benefits of therapy relative to trial outcomes.

A 2011 clinical trial^14^ demonstrated increasing mortality risk with increasing severity of illness based on INTERMACS profiles. Specifically, that study found that patients with less acute illness (ie, profiles 4-7) had superior survival and shorter length of stay compared with patients with more acute illness (ie, profiles 1 and 2). As expected, mortality in our study was highest for patients with INTERMACS profile 1, followed by profile 2, but there was no significant difference between profile 3 and profiles 4 through 7. Interestingly, 1-year mortality rates among patients who were trial-eligible with INTERMACS Profiles 1, 2, 3, and 4 through 7 in this study were all comparable with 1-year survival found in ENDURANCE II (16.9% in the HeartWare LVAD group and 17.8% in the HMII control group)^5^ and MOMENTUM 3 (15.9% for the HMII control group).^8^ These data for mortality by trial eligibility and profile number in the limited and comprehensive analyses suggest that ineligibility criteria are associated with prognosis, independent of INTERMACS profile.

Our findings may also have important implications for regulators and payers. To provide estimates of benefit closer to real-world experience, regulators, such as the FDA, could limit the number and types of exclusion criteria used in clinical trials. Clinical registries could be designed to more closely follow patients receiving commercial durable LVAD devices who were ineligible for clinical trials to understand outcomes in these patients, potentially by mandating registry arms for patients who are trial-ineligible. Regulators may wish to incorporate these data to support coverage decisions, develop guidelines and decision support tools, monitor postmarket safety through more robust postmarket approval studies, and develop innovative treatment approaches, as required by the 21st Century Cures Act of 2016.^15,16^ Payers could also consider our findings in future payment coverage determinations. For instance, CMS originally based reimbursement for LVAD therapy under the National Coverage Determination on inclusion criteria from the Randomized Evaluation of Mechanical Assistance for the Treatment of Congestive Heart Failure (REMATCH) trial.^10,17^ Incorporating trial criteria in coverage determinations may be associated with reimbursement of a smaller population, which may be associated with reduced incentives among clinicians of using durable LVAD therapy in patients at higher risk of mortality.

However, with a 1-year mortality of approximately 25%, patients who were trial-ineligible in this study may still benefit from LVAD implantation over medical therapy. Connecting exclusion criteria to reimbursement could also be associated with increased incentives among trialists to limit exclusion criteria in trials to expand the patient population eligible for reimbursement, which may provide more real-world estimates of benefit and may ultimately benefit a larger group of patients undergoing durable LVAD therapy instead of medical therapy, which is associated with an extremely poor prognosis. More informed regulatory and payment decisions are critical for optimizing both patient outcomes and the societal burden of durable LVAD therapy.

### Limitations

This study has several limitations. First, participants in the MOMENTUM 3 trial receiving the HM3 device and patients receiving devices not approved by the FDA were not included in the analysis. While this study found worse outcomes for recipients of HMII or HeartWare LVAD who were trial-ineligible, the most recent INTERMACS report found that HM3 outcomes were comparable between patients in MOMENTUM 3 and those in INTERMACS.^1^ Although recipients of HM3 were not included in this study, patients receiving HMII as part of the control device group in MOMENTUM 3 were included in INTERMACS and are represented here. Additionally, exclusion criteria among major LVAD trials^3,4,5,6,7,8,9,18^ have remained relatively consistent, and our intention in choosing MOMENTUM 3 criteria was to most accurately inform present-day device selection. Furthermore, when these trial criteria were applied temporally within this study population, real-world recipients of LVAD were just as likely to be ineligible for trial in 2012 as in 2017. Second, not every trial criterion from MOMENTUM 3 could be implemented through INTERMACS. However, 2 sets of criteria (limited and comprehensive) were used to together provide a full assessment of trial eligibility in this population. Third, this analysis focuses exclusively on mortality, while other outcomes, such as disabling stroke, permanent dialysis, and patient-reported outcomes, including quality of life, must be considered to fully characterize the utility and drawbacks of durable LVAD therapy.

## Conclusions

The findings of this cohort study suggest that nearly one-half of real-world LVAD recipients did not meet current trial criteria. This ineligibility for trial inclusion was associated with increased mortality, and prior or ongoing MCS, elevated bilirubin or creatinine levels, and low albumin or prealbumin levels were independently associated with mortality. Patients meeting any 3 or more ineligibility criteria had more than 2-fold the risk of mortality compared with patients who were trial-eligible. Importantly, these data demonstrated an association between trial ineligibility criteria and postimplant prognosis, independent of baseline heart failure severity. However, these patients who were trial-ineligible may still be considered for potential LVAD therapy by comparing prognosis with medical management alone. Clinical trialists, regulators, and payers should consider the generalizability and prognostic value of individual eligibility criteria to further refine LVAD trial populations. Likewise, clinicians should use trial criteria to inform clinical decision-making when evaluating LVAD candidates and prognosticating in real-world practice.

## Notes

Supplement. **eTable 1. **INTERMACS Variables Used to Define Trial Eligibility for Primary (Limited) and Sensitivity (Comprehensive) Analyses **eTable 2. **INTERMACS Mapped Variable Values Used to Define Trial Eligibility for Primary (Limited) and Sensitivity (Comprehensive) Analyses **eTable 3. **Cox Proportional Hazards Model for Overall Mortality by Trial Eligibility Status (Full Model) **eTable 4. **Distribution of Inclusion and Exclusion Criteria Determining Trial-Ineligible Status in Limited and Comprehensive Analyses **eTable 5. **Cox Proportional Hazards Model for Overall Mortality by Specific Exclusion Criterion, Adjusted for Baseline Patient Characteristics **eTable 6. **Cox Proportional Hazards Model for Overall Mortality by Specific Ineligibility Criterion Among Patients Meeting at Least 3 Criteria, Adjusted for Baseline Patient Characteristics **eFigure 1. **Study Population **eFigure 2. **Ineligibility by Year **eFigure 3. **Distribution of Ineligibility Criteria **eFigure 4. **Frequency of Ineligibility Criteria **eFigure 5. **Cumulative Incidence of Mortality With Transplant as a Competing Risk **eFigure 6. **Mortality by Eligibility Status Stratified by Device Type **eFigure 7. **Mortality by INTERMACS Profile **eFigure 8. **Mortality by Eligibility Stratified by INTERMACS Profile for Limited Criteria **eFigure 9. **Mortality by Eligibility Stratified by INTERMACS Profile for Comprehensive Criteria
