# Error in Figure

**DOI:** 10.1001/jamanetworkopen.2021.18963

**Published:** 2021-06-21

In the Original Investigation titled “Assessment of Mortality Among Durable Left Ventricular Assist Device Recipients Ineligible for Clinical Trials,”^1^ published January 8, 2021, the key for Figure 2 mistakenly transposed the color for 1 ineligibility criterion with the color for 3 or more ineligibility criteria. This article has been corrected.^1^
