# Supplementary material for: Assessment of Mortality Among Durable Left Ventricular Assist Device Recipients Ineligible for Clinical Trials
Source: JAMA Netw Open. 2021 Jan 8;4(1):e2032865. doi: 10.1001/jamanetworkopen.2020.32865 (PMC7794668; doi:10.1001/jamanetworkopen.2020.32865)
Supplement: Supplement. — eTable 1. INTERMACS Variables Used to Define Trial Eligibility for Primary (Limited) and Sensitivity (Comprehensive) Analyses eTable 2. INTERMACS Mapped Variable Values Used to Define Trial Eligibility for Primary (Limited) and Sensitivity (Comprehensive) Analyses eTable 3. Cox Proportional Hazards Model for Overall Mortality by Trial Eligibility Status (Full Model) eTable 4. Distribution of Inclusion and Exclusion Criteria Determining Trial-Ineligible Status in Limited and Comprehensive Analyses eTable 5. Cox Proportional Hazards Model for Overall Mortality by Specific Exclusion Criterion, Adjusted for Baseline Patient Characteristics eTable 6. Cox Proportional Hazards Model for Overall Mortality by Specific Ineligibility Criterion Among Patients Meeting at Least 3 Criteria, Adjusted for Baseline Patient Characteristics eFigure 1. Study Population eFigure 2. Ineligibility by Year eFigure 3. Distribution of Ineligibility Criteria eFigure 4. Frequency of Ineligibility Criteria eFigure 5. Cumulative Incidence of Mortality With Transplant as a Competing Risk eFigure 6. Mortality by Eligibility Status Stratified by Device Type eFigure 7. Mortality by INTERMACS Profile eFigure 8. Mortality by Eligibility Stratified by INTERMACS Profile for Limited Criteria eFigure 9. Mortality by Eligibility Stratified by INTERMACS Profile for Comprehensive Criteria [file jamanetwopen-e2032865-s001.pdf]

Supplemental Online Content

Brescia AA, Watt TMF, Pagani FD, et al; Michigan Congestive Heart Failure Investigators. Assessment of mortality among durable left ventricular assist device recipients ineligible for clinical trials. *JAMA Netw Open*. 2021;4(1):e2032865. doi: 10.1001/jamanetworkopen.2020.32865

eTable 1. INTERMACS Variables Used to Define Trial Eligibility for Primary (Limited) and Sensitivity (Comprehensive) Analyses

eTable 2. INTERMACS Mapped Variable Values Used to Define Trial Eligibility for Primary (Limited) and Sensitivity (Comprehensive) Analyses

eTable 3. Cox Proportional Hazards Model for Overall Mortality by Trial Eligibility Status (Full Model)

eTable 4. Distribution of Inclusion and Exclusion Criteria Determining Trial-Ineligible Status in Limited and Comprehensive Analyses

eTable 5. Cox Proportional Hazards Model for Overall Mortality by Specific Exclusion Criterion, Adjusted for Baseline Patient Characteristics

eTable 6. Cox Proportional Hazards Model for Overall Mortality by Specific Ineligibility Criterion Among Patients Meeting at Least 3 Criteria, Adjusted for Baseline Patient Characteristics

eFigure 1. Study Population

eFigure 2. Ineligibility by Year

eFigure 3. Distribution of Ineligibility Criteria

eFigure 4. Frequency of Ineligibility Criteria

eFigure 5. Cumulative Incidence of Mortality With Transplant as a Competing Risk

eFigure 6. Mortality by Eligibility Status Stratified by Device Type

eFigure 7. Mortality by INTERMACS Profile

eFigure 8. Mortality by Eligibility Stratified by INTERMACS Profile for Limited Criteria

eFigure 9. Mortality by Eligibility Stratified by INTERMACS Profile for Comprehensive Criteria

This supplemental material has been provided by the authors to give readers additional information about their work.

eTable 1. INTERMACS Variables Used to Define Trial Eligibility for Primary (Limited) and Sensitivity (Comprehensive) Analyses

Interagency Registry for Mechanically Assisted Circulatory Support (INTERMACS) variables used to define trial eligibility for primary (limited) and sensitivity (comprehensive) analyses. In the comprehensive analysis, concomitant RVAD with LVAD implantation was considered a planned procedure and therefore, all patients receiving concomitant RVAD in INTERMACS were considered trial-ineligible by virtue of meeting the "planned biventricular support" exclusion criterion. Variable labels are shown, while mapped variable values are shown in **eTable 2**. Empty cells indicate criteria that were not applied to INTERMACS patients. BSA, body surface area; FEV1, forced expiratory volume in one second; FVC, forced vital capacity; INR, International Normalized Ratio; NYHA, New York Heart Association; LVAD, left ventricular assist device; LVEF, left ventricular ejection fraction; PVR, pulmonary vascular resistance.

**MOMENTUM 3 Trial Criteria**	**Primary Analysis (Limited Criteria)**	**Sensitivity Analysis (Comprehensive Criteria)**
**INCLUSION CRITERIA**		
Age >=18 years old	Age at Implant < 18	Age at Implant < 18
BSA >=1.2 m^2	Body Surface Area < 1.2 meters squared	Body Surface Area < 1.2 meters squared
NYHA Class III	NYHA Class I or II	NYHA Class I or II
LVEF <=25%	LVEF >50%, 40-49%, or 30-39%	LVEF >50%, 40-49%, or 30-39%
Inotrope Dependent or CI<2.2 L/min/m^2	IV Inotrope Therapy (No); Cardiac Index	IV Inotrope Therapy (No); Cardiac Index
Subject or legal representative has signed Informed Consent Form		
Females of child bearing age must agree to use adequate contraception		
**EXCLUSION CRITERIA**		
Concomitant Procedures		
Patient has moderate to severe aortic insufficiency without plans for correction	Aortic Insufficiency Moderate or Severe; Concomitant Valve Surgery Aortic Repair: Valve Closure (No) or No Valve Closure (No) or Valve Surgery Aortic Replacement Biological (No)	Aortic Insufficiency Moderate or Severe; Concomitant Valve Surgery Aortic Repair: Valve Closure (No) or No Valve Closure (No) or Valve Surgery Aortic Replacement Biological (No)
Presence of mechanical aortic cardiac valve that will not be either converted to bioprosthetic or oversewn	Aortic Insufficiency Moderate or Severe and Valve Surgery Aortic Replacement Mechanical (Yes)	Aortic Insufficiency Moderate or Severe and Valve Surgery Aortic Replacement Mechanical (Yes)
Non-Compliance or Psychosocial Issues		
Psychiatric disease/disorder, irreversible cognitive dysfunction or psychosocial issues that are likely to impair compliance with the study protocol and LVAD management		Severe Depression (Yes) or Other Major Psychiatric Diagnosis (Yes) or Limited Cognitive Understanding (Yes) or Reported Non-Compliance (Yes)
Nutrition		

© 2021 Brescia AA et al. *JAMA Network Open.*

Pre albumin < 150 mg/L (15mg/dL) or Albumin < 30g/L (3 g/dL) (if only one available); pre albumin < 150 mg/L (15mg/dL) and Albumin < 30g/L (3 g/dL) (if both available)	Pre-albumin < 15 mg/dl or Albumin < 3 g/dL (if only one present) or Pre-albumin < 15 mg/dl and Albumin < 3 g/dL (if both present) (if both are missing, the patient was treated as eligible)	Pre-albumin < 15 mg/dl or Albumin < 3 g/dL (if only one present) or Pre-albumin < 15 mg/dl and Albumin < 3 g/dL (if both present) (if both are missing, the patient was treated as eligible)
Liver Function		
INR > 2.0 not due to anticoagulation therapy	INR > 2	INR > 2
Total bilirubin >43 umol/L (2.5 mg/dl)	Total Bilirubin > 2.5 mg/dL	Total Bilirubin > 2.5 mg/dL
Shock liver or biopsy proven liver cirrhosis		Liver Dysfunction (Yes)
Renal Function		
Serum Creatinine >221umol/L (2.5 mg/dl) or the need for chronic renal replacement therapy	Creatinine > 2.5 mg/dL or Intervention – Ultrafiltration Within Last 48 Hours (Yes)	Creatinine > 2.5 mg/dL or Intervention – Ultrafiltration Within Last 48 Hours (Yes)
Coagulation		
Platelet count < 100,000 x 103/L (< 100,000/ml)	Platelets (x10/uL) < 100	Platelets (x10/uL) < 100
Patient has known hypo or hyper coagulable states such as disseminated intravascular coagulation and heparin induced thrombocytopenia		Chronic Coagulopathy (Yes)
Pulmonary Vascular Hypertension		
Fixed pulmonary hypertension with a most recent PVR = 8 Wood units that is unresponsive to pharmacologic intervention		Pulmonary Vascular Resistance Using Cardiac Output > or = 8
Major Infection		
Presence of an active, uncontrolled infection		Events This Hospitalization – Major Infection (Yes)
Right Ventricular Failure		
Planned Bi-VAD support prior to enrollment	Intervention – RVAD Placement Within Last 48 Hours (Yes)	Intervention – RVAD Placement Within Last 48 Hours (Yes) or Bi-VAD (Yes) or Concomitant Surgery – RVAD Implant (Yes)
Pulmonary Disease		
History of severe chronic obstructive pulmonary disease defined by FEV1/FVC < 0.7, and FEV1 <50% predicted		Pulmonary Disease (Yes)
Peripheral Vascular Disease		
Significant peripheral vascular disease (PVD) accompanied by rest pain or extremity ulceration		Peripheral Vascular Disease (Yes)
History of confirmed, untreated Abdominal Aortic Aneurysm		Same code as above, so AAA were not included in our analysis

© 2021 Brescia AA et al. *JAMA Network Open.*

(AAA) > 5 cm in diameter within 6 months of enrollment		
Cerebrovascular Disease		
History of stroke within 90 days prior to enrollment, or a history of cerebrovascular disease with significant (> 80%) uncorrected carotid artery stenosis		Major Stroke (Yes) or Other Cerebrovascular Disease (Yes)
Transplant History		
History of any organ transplant	Previous Cardiac Operation – Previous Heart Transplant (Yes) or History Bone Marrow Transplant (Yes)	Previous Cardiac Operation – Previous Heart Transplant (Yes) or History Bone Marrow Transplant (Yes)
Prior Mechanical Circulatory Support		
Existence of ongoing mechanical circulatory support (MCS) other than intra-aortic balloon pump	Intervention – ECMO Within Last 48 Hours (Yes) or Intervention – LVAD Within Last 48 Hours (Yes) or Intervention – RVAD Within Last 48 Hours (Yes) or Intervention – Total Artificial Heart Within Last 48 Hours (Yes) or Concomitant Surgery – ECMO Decannulation (Yes)	Intervention – ECMO Within Last 48 Hours (Yes) or Intervention – LVAD Within Last 48 Hours (Yes) or Intervention – RVAD Within Last 48 Hours (Yes) or Intervention – Total Artificial Heart Within Last 48 Hours (Yes) or Concomitant Surgery – ECMO Decannulation (Yes)
Etiology of Disease		
Etiology of heart failure due to or associated with uncorrected thyroid disease, obstructive cardiomyopathy, pericardial disease, amyloidosis or restrictive cardiomyopathy	Primary Diagnosis Restrictive Myopathy (Yes) or Primary Diagnosis Hypertrophic Cardiomyopathy (Yes) or Restrictive Myopathy – Amyloidosis (Yes) or Restrictive Myopathy – Endocardial Fibrosis (Yes) or Restrictive Myopathy – Idiopathic (Yes) or Restrictive Myopathy – Other (Yes) or Restrictive Myopathy Secondary to Radiation or Chemotherapy (Yes)	Primary Diagnosis Restrictive Myopathy (Yes) or Primary Diagnosis Hypertrophic Cardiomyopathy (Yes) or Restrictive Myopathy – Amyloidosis (Yes) or Restrictive Myopathy – Endocardial Fibrosis (Yes) or Restrictive Myopathy – Idiopathic (Yes) or Restrictive Myopathy – Other (Yes) or Restrictive Myopathy Secondary to Radiation or Chemotherapy (Yes)
Other Exclusions		
Technical obstacles which pose an inordinately high surgical risk, in the judgment of the investigator		Intervention – Aneursyomectomy Within Last 48 Hours (Yes) or Intervention – Aortic Valve Replacement/Repair Within Last 48 Hours (Yes) or Intervention – Coronary Artery Bypass Grafting Within Last 48 Hours (Yes) or Intervention – Congential Cardiac Surgery Within Last 48 Hours (Yes) or Intervention – Mitral Valve Replacement/Repair Within Last 48 Hours
Positive pregnancy test if of childbearing potential		
Intolerance to anticoagulant or antiplatelet therapies or any other peri/post-operative therapy the investigator will require based upon patients' health status		
Participation in any other clinical investigation that is likely to confound study results or affect the study		

Any condition other than HF that could limit survival to less than 24 months		

© 2021 Brescia AA et al. *JAMA Network Open.*

eTable 2. INTERMACSMapped Variable Values Used to Define Trial Eligibility for Primary (Limited) and Sensitivity (Comprehensive) Analyses

Interagency Registry for Mechanically Assisted Circulatory Support (INTERMACS) mapped variable values used to define trial eligibility for primary (limited) and sensitivity (comprehensive) analyses. Empty cells indicate criteria that were not applied to INTERMACS patients. BSA, body surface area; FEV1, forced expiratory volume in one second; FVC, forced vital capacity; INR, International Normalized Ratio; NYHA, New York Heart Association; LVAD, left ventricular assist device; LVEF, left ventricular ejection fraction; PVR, pulmonary vascular resistance.

MOMENTUM 3 Trial Criteria	Primary Analysis (Limited Criteria)	Sensitivity Analysis (Comprehensive Criteria)
**INCLUSION CRITERIA**		
Age >=18 years old	AGE<18	AGE<18
BSA >=1.2 m^2	BSA<1.2	BSA<1.2
NYHA Class III	NYHA=1 or NYHA=2	NYHA=1 or NYHA=2
LVEF <=25%	LVEF=1, 2, or 3	LVEF=1, 2, or 3
Inotrope Dependent or CI<2.2 L/min/m^2	IV_INO_THERAPY=0 and CARDIAC_INDEX>2.2	IV_INO_THERAPY=0 and CARDIAC_INDEX>2.2
Subject or legal representative has signed Informed Consent Form		
Females of child bearing age must agree to use adequate contraception		
**EXCLUSION CRITERIA**		
Concomitant Procedures		
Patient has moderate to severe aortic insufficiency without plans for correction	AORTIC_INSUFFICIENCY=2 or AORTIC_INSUFFICIENCY=3 and CONCOM_SURG_AVS_C=0 or CONCOM_SURG_AVS_NC=0 or CONCOM_SURG_AVS_REP_BIO=0	AORTIC_INSUFFICIENCY=2 or AORTIC_INSUFFICIENCY=3 and CONCOM_SURG_AVS_C=0 or CONCOM_SURG_AVS_NC=0 or CONCOM_SURG_AVS_REP_BIO=0
Presence of mechanical aortic cardiac valve that will not be either converted to bioprosthetic or oversewn	AORTIC_INSUFFICIENCY=2 or AORTIC_INSUFFICIENCY=3 and CONCOM_SURG_AVS_REP_MECH=1	AORTIC_INSUFFICIENCY=2 or AORTIC_INSUFFICIENCY=3 and CONCOM_SURG_AVS_REP_MECH=1
Non-Compliance or Psychosocial Issues		
Psychiatric disease/disorder, irreversible cognitive dysfunction or psychosocial issues that are likely to impair compliance with the study protocol and LVAD management		CC2_SEVERE_DEPRESSION_M=1 or CC2_OTH_MAJOR_PSYCH_DIAG_M=1 or CC2_LTD_COG_UNDERSTND_M=1 or CC2_RPTD_NON_COMPLIANCE_M=1
Nutrition		

Pre albumin < 150 mg/L (15mg/dL) or Albumin < 30g/L (3 g/dL) (if only one available); pre albumin < 150 mg/L (15mg/dL) and Albumin < 30g/L (3 g/dL) (if both available)	PRE_ALBUMIN_MG_DL < 15 mg/dl or ALBUMIN_G_DL < 3 g/dL (if only one present) or PRE_ALBUMIN_MG_DL < 15 mg/dl and ALBUMIN_G_DL < 3 g/dL (if both present)<br>(if missing, treated as eligible)	PRE_ALBUMIN_MG_DL < 15 mg/dl or ALBUMIN_G_DL < 3 g/dL (if only one present) or PRE_ALBUMIN_MG_DL < 15 mg/dl and ALBUMIN_G_DL < 3 g/dL (if both present)<br>(if missing, treated as eligible)
Liver Function		
INR > 2.0 not due to anticoagulation therapy	INR>2	INR>2
Total bilirubin >43 umol/L (2.5 mg/dl)	BILI_TOTAL_MG_DL>2.5	BILI_TOTAL_MG_DL>2.5
Shock liver or biopsy proven liver cirrhosis		CC2_LIVER_DYSFUNCTION_M=1
Renal Function		
Serum Creatinine >221umol/L (2.5 mg/dl) or the need for chronic renal replacement therapy	CREAT_MG_DL>2.5 or INTERVENTION_48_HRS_ULTRAFILT=1	CREAT_MG_DL>2.5 or INTERVENTION_48_HRS_ULTRAFILT=1
Coagulation		
Platelet count < 100,000 x 103/L (< 100,000/ml)	PLATELET_X10_3_UL<100	PLATELET_X10_3_UL<100
Patient has known hypo or hyper coagulable states such as disseminated intravascular coagulation and heparin induced thrombocytopenia		CC2_CHRONIC_COAGULOPATHY=1
Pulmonary Vascular Hypertension		
Fixed pulmonary hypertension with a most recent PVR = 8 Wood units that is unresponsive to pharmacologic intervention		PVR_CO>=8
Major Infection		
Presence of an active, uncontrolled infection		EVENT_HOSP_MAJOR_INF=1
Right Ventricular Failure		
Planned Bi-VAD support prior to enrollment	INTERVENTION_48_HRS_RVAD=1	INTERVENTION_48_HRS_RVAD=1 or BIVAD=1 or CONCOM_SURG_RVAD_IMPLANT=1
Pulmonary Disease		
History of severe chronic obstructive pulmonary disease defined by FEV1/FVC < 0.7, and FEV1 <50% predicted		CC2_PULMONARY_DISEASE_M=1
Peripheral Vascular Disease		
Significant peripheral vascular disease (PVD) accompanied by rest pain or extremity ulceration		CC2_PERIPH_VASC_DISEASE_M=1
History of confirmed, untreated Abdominal Aortic Aneurysm (AAA) > 5 cm in diameter within 6 months of enrollment		Same code as above, so AAA were not included in our analysis

© 2021 Brescia AA et al. *JAMA Network Open.*

Cerebrovascular Disease		
History of stroke within 90 days prior to enrollment, or a history of cerebrovascular disease with significant (> 80%) uncorrected carotid artery stenosis		CC2_MAJOR_STROKE_M=1 or CC2_OTH_CEREBROVASC_DISEASE_M=1
Transplant History		
History of any organ transplant	PREV_CARDIAC_OPER_PREV_TRANS=1 or CC2_HIST_BONE_MARROW_TX=1	PREV_CARDIAC_OPER_PREV_TRANS=1 or CC2_HIST_BONE_MARROW_TX=1
Prior Mechanical Circulatory Support		
Existence of ongoing mechanical circulatory support (MCS) other than intra-aortic balloon pump	INTERVENTION_48_HRS_ECMO=1 or INTERVENTION_48_HRS_LVAD=1 or INTERVENTION_48_HRS_RVAD=1 or INTERVENTION_48_HRS_TAH=1 or CONCOM_SURG_ECMO_DECANN=1	INTERVENTION_48_HRS_ECMO=1 or INTERVENTION_48_HRS_LVAD=1 or INTERVENTION_48_HRS_RVAD=1 or INTERVENTION_48_HRS_TAH=1 or CONCOM_SURG_ECMO_DECANN=1
Etiology of Disease		
Etiology of heart failure due to or associated with uncorrected thyroid disease, obstructive cardiomyopathy, pericardial disease, amyloidosis or restrictive cardiomyopathy	PRIMARY_DGN_REST_MYO_OSTXT=1 or PRIMARY_DGN=13, 14, 15, 16, 17, 19	PRIMARY_DGN_REST_MYO_OSTXT=1 or PRIMARY_DGN=13, 14, 15, 16, 17, 19
Other Exclusions		
Technical obstacles which pose an inordinately high surgical risk, in the judgment of the investigator		INTERVENTION_48_HRS_ANEURS=1 or INTERVENTION_48_HRS_AVR=1 or INTERVENTION_48_HRS_CABG=1 or INTERVENTION_48_HRS_CON_CAR_SUR=1 or INTERVENTION_48_HRS_MVR=1
Positive pregnancy test if of childbearing potential		
Intolerance to anticoagulant or antiplatelet therapies or any other peri/post-operative therapy the investigator will require based upon patients' health status		
Participation in any other clinical investigation that is likely to confound study results or affect the study		
Any condition other than HF that could limit survival to less than 24 months		

© 2021 Brescia AA et al. *JAMA Network Open.*

eTable 3. Cox Proportional Hazards Model for Overall Mortality by Trial Eligibility Status (Full Model)

All risk-adjustment covariates are included to evaluate the overall mortality for trial-ineligible versus trial-eligible patients. Individual hospital was included as a random effect through a shared frailty model.

	Limited Analysis		Comprehensive Analysis	
**Variable**	**Hazard Ratio (95% CI)**	***P*-value**	**Hazard Ratio (95% CI)**	***P*-value**
Trial-ineligible (vs. trial-eligible)	1.29 (1.21–1.37)	<0.001	1.42 (1.33–1.52)	<0.001
HVAD (vs. HeartMate II) device	1.42 (1.30–1.56)	<0.001	1.43 (1.30–1.57)	<0.001
Age, per year	1.02 (1.02–1.03)	<0.001	1.02 (1.02–1.03)	<0.001
Female vs. male sex	1.15 (1.06–1.24)	<0.001	1.15 (1.06–1.24)	<0.001
Body mass index (higher)	1.01 (1.01–1.01)	<0.001	1.01 (1.01–1.01)	<0.001
Blood type 0	0.98 (0.95–1.01)	0.183	0.98 (0.95–1.01)	0.191
White race	1.09 (1.01–1.17)	0.024	1.09 (1.01–1.17)	0.021
Implantable cardioverter defibrillator	1.22 (1.12–1.33)	<0.001	1.22 (1.12–1.33)	<0.001
INTERMACS Profile (Reference: 4-7)				
Profile 1	1.67 (1.49–1.86)	<0.001	1.63 (1.46–1.83)	<0.001
Profile 2	1.30 (1.18–1.43)	<0.001	1.28 (1.16–1.41)	<0.001
Profile 3	1.11 (1.01–1.23)	0.031	1.11 (1.01–1.23)	0.033
Bridge-to-transplant listed	0.68 (0.61–0.75)	<0.001	0.69 (0.63–0.77)	<0.001
Bridge-to-transplant likely	0.65 (0.59–0.73)	<0.001	0.66 (0.60–0.74)	<0.001
History of coronary artery bypass grafting	1.29 (1.20–1.39)	<0.001	1.28 (1.19–1.38)	<0.001
History of valve surgery	1.15 (1.04–1.28)	0.010	1.16 (1.04–1.29)	0.008
Concomitant cardiac surgery	1.25 (1.17–1.33)	<0.001	1.24 (1.16–1.32)	<0.001
Implant Year				
2012	Reference		Reference	
2013	0.97 (0.87–1.08)	0.560	0.98 (0.88–1.08)	0.664
2014	0.96 (0.86–1.07)	0.487	0.96 (0.87–1.07)	0.500
2015	0.98 (0.88–1.10)	0.737	0.99 (0.88–1.10)	0.794
2016	0.84 (0.75–0.96)	0.008	0.85 (0.75–0.96)	0.009
2017	0.89 (0.75–1.05)	0.175	0.89 (0.75–1.05)	0.173

© 2021 Brescia AA et al. *JAMA Network Open.*

eTable 4. Distribution of Inclusion and Exclusion Criteria Determining Trial-Ineligible Status in Limited and Comprehensive Analyses

Distribution of inclusion and exclusion criteria determining trial-ineligible status in both the limited and comprehensive analyses. Percentages are calculated from total population n=14,679. The highest number of ineligibility criteria per patient was 6 in the limited analysis and 9 in the comprehensive analysis.

**Limited Criteria Analysis**				**Comprehensive Criteria Analysis**			
**Number of trial ineligibility criteria met**	**Number of inclusions not met**	**Number of exclusions met**	**n (%)**	**Number of trial ineligibility criteria met**	**Number of inclusions not met**	**Number of exclusions met**	**n (%)**
Zero (trial-eligible)	0	0	8250 (56.2)	Zero (trial-eligible)	0	0	6791 (46.3)
One	1	0	1001 (6.8)	One	1	0	842 (5.7)
	0	1	3225 (22.0)		0	1	3721 (25.3)
Two	2	0	47 (0.3)	Two	2	0	41 (0.3)
	1	1	332 (2.3)		1	1	402 (2.7)
	0	2	1063 (7.2)		0	2	1572 (10.7)
Three	3	0	1 (0)	Three	3	0	1 (0)
	2	1	11 (0.1)		2	1	16 (0.1)
	1	2	89 (0.6)		1	2	133 (0.9)
	0	3	430 (2.9)		0	3	632 (4.3)
Four	4	0	0	Four	4	0	0
	3	1	1 (0)		3	1	0
	2	2	4 (0)		2	2	4 (0)
	1	3	34 (0.2)		1	3	67 (0.5)
	0	4	135 (0.9)		0	4	281 (1.9)
Five	4	1	0	Five	4	1	0
	3	2	1 (0)		3	2	1 (0)
	2	3	1 (0)		2	3	2 (0)
	1	4	14 (0.1)		1	4	16 (0.1)
	0	5	33 (0.2)		0	5	108 (0.7)
Six	4	2	0	Six	4	2	0
	3	3	0		3	3	1 (0)
	2	4	0		2	4	0
	1	5	3 (0)		1	5	8 (0.1)
	0	6	4 (0)		0	6	28 (0.2)

© 2021 Brescia AA et al. *JAMA Network Open.*

	Seven	4	3	0
		3	4	0
		2	5	0
		1	6	4 (0)
		0	7	5 (0)
	Eight	4	4	0
		3	5	0
		2	6	0
		1	7	1 (0)
		0	8	1 (0)
	Nine	4	5	0
		3	6	0
		2	7	0
		1	8	0
		0	9	1 (0)

© 2021 Brescia AA et al. *JAMA Network Open.*

eTable 5. Cox Proportional Hazards Model for Overall Mortality by Specific Ineligibility Criterion, Adjusted for Baseline Patient Characteristics

Cox proportional hazards model for overall mortality by specific exclusion criterion, adjusted for baseline patient characteristics. A separate Cox model was created for each exclusion criterion and all models adjusted for age, sex, body mass index, blood type O, white race, implantable cardioverter defibrillator presence, Interagency Registry for Mechanically Assisted Circulatory Support (INTERMACS) patient Profile, bridge-to-transplant status, prior coronary artery bypass or valve surgery, concomitant cardiac surgery, device type, and year of implant. The reference group for all Cox hazard ratios (HR) is trial-eligible patients. AI, aortic insufficiency; CI, confidence interval; INR, International Normalized Ratio; LVEF, left ventricular ejection fraction; MCS, mechanical circulatory support.

	**Specific exclusion present (with or without others)**			**Only criterion present**			**This criterion plus ≥1 other criterion present**		
	n	***HR (95% CI)***	***p-value***	***n***	***HR (95% CI)***	***p-value***	***n***	***HR (95% CI)***	***p-value***
Inclusion criteria not met									
Inotrope dependent or cardiac index <2.2 L/min/m^2	759	0.99 (0.86–1.14)	0.89	513	0.96 (0.80–1.15)	0.68	246	1.36 (1.08–1.72)	0.009
LVEF ≤25%	621	1.23 (1.07–1.41)	0.003	348	1.10 (0.90–1.34)	0.36	273	1.90 (1.56–2.31)	<0.001
Exclusion criteria met									
Low albumin/pre-albumin	2281	1.24 (1.14–1.35)	<0.001	1064	1.18 (1.05–1.33)	0.007	1217	1.69 (1.50–1.90)	<0.001
Platelets <100,000/mL	1369	1.36 (1.23–1.50)	<0.001	438	1.13 (0.94–1.35)	0.20	931	1.81 (1.59–2.06)	<0.001
Bilirubin >2.5 mg/dL	1293	1.53 (1.38–1.69)	<0.001	523	1.39 (1.17–1.66)	<0.001	770	1.99 (1.74–2.27)	<0.001
Prior/ongoing MCS (other than intra-aortic balloon pump)	973	1.54 (1.35–1.75)	<0.001	197	1.63 (1.23–2.16)	0.001	776	2.05 (1.73–2.42)	<0.001
Creatinine >2.5 mg/dl	691	1.50 (1.32–1.70)	<0.001	319	1.42 (1.17–1.72)	<0.001	372	2.13 (1.79–2.53)	<0.001
INR >2.0 and not on anticoagulation therapy	590	1.05 (0.90–1.22)	0.52	301	0.92 (0.73–1.16)	0.50	289	1.71 (1.39–2.10)	<0.001
Moderate to Severe AI without plan to correct it	501	0.99 (0.84–1.15)	0.86	252	0.88 (0.69–1.11)	0.28	249	1.49 (1.20–1.83)	<0.001

© 2021 Brescia AA et al. *JAMA Network Open.*

eTable 6. Cox Proportional Hazards Model for Overall Mortality by Specific Ineligibility Criterion Among Patients Meeting at Least 3 Criteria, Adjusted for Baseline Patient Characteristics

Cox proportional hazards model for overall mortality by specific ineligibility criterion, adjusted for baseline patient characteristics. A separate Cox model was created for each combination and all models adjusted for age, sex, body mass index, blood type O, white race, implantable cardioverter defibrillator presence, Interagency Registry for Mechanically Assisted Circulatory Support (INTERMACS) patient Profile, bridge-to-transplant status, prior coronary artery bypass or valve surgery, concomitant cardiac surgery, device type, and year of implant. The reference group for all Cox hazard ratios (HR) is trial-eligible patients. AI, aortic insufficiency; CI, confidence interval; INR, International Normalized Ratio; LVEF, left ventricular ejection fraction; MCS, mechanical circulatory support.

Ineligibility criteria met	***n***	***HR (95% CI)***	***p-value***
Low albumin/pre-albumin, platelets <100,000/mL, and prior/ongoing MCS	228	2.27 (1.77–2.93)	<0.001
Low albumin/pre-albumin, platelets <100,000/mL, and bilirubin >2.5 mg/dL	153	2.41 (1.83–3.16)	<0.001
Platelets <100,000/mL, bilirubin >2.5 mg/dL, and prior/ongoing MCS	134	3.08 (2.31–4.12)	<0.001
Low albumin/pre-albumin, bilirubin >2.5 mg/dL, and prior/ongoing MCS	128	2.81 (2.09–3.78)	<0.001
Platelets <100,000/mL, bilirubin >2.5 mg/dL, and creatinine >2.5 mg/dL	53	3.53 (2.43–5.12)	<0.001
Low albumin/pre-albumin, platelets <100,000/mL, and creatinine >2.5 mg/dL	50	2.61 (1.70–4.01)	<0.001
Platelets <100,000/mL, prior/ongoing MCS, and creatinine >2.5 mg/dL	48	2.81 (1.83–4.30)	<0.001
Low albumin/pre-albumin, prior/ongoing MCS, and creatinine >2.5 mg/dL	45	2.72 (1.75–4.23)	<0.001
Bilirubin >2.5 mg/dL, prior/ongoing MCS, and creatinine >2.5 mg/dL	39	2.58 (1.62–4.11)	<0.001
Low albumin/pre-albumin, bilirubin >2.5 mg/dL, and creatinine >2.5 mg/dL	38	3.98 (2.58–6.14)	<0.001

© 2021 Brescia AA et al. *JAMA Network Open.*

eFigure 1. Study Population

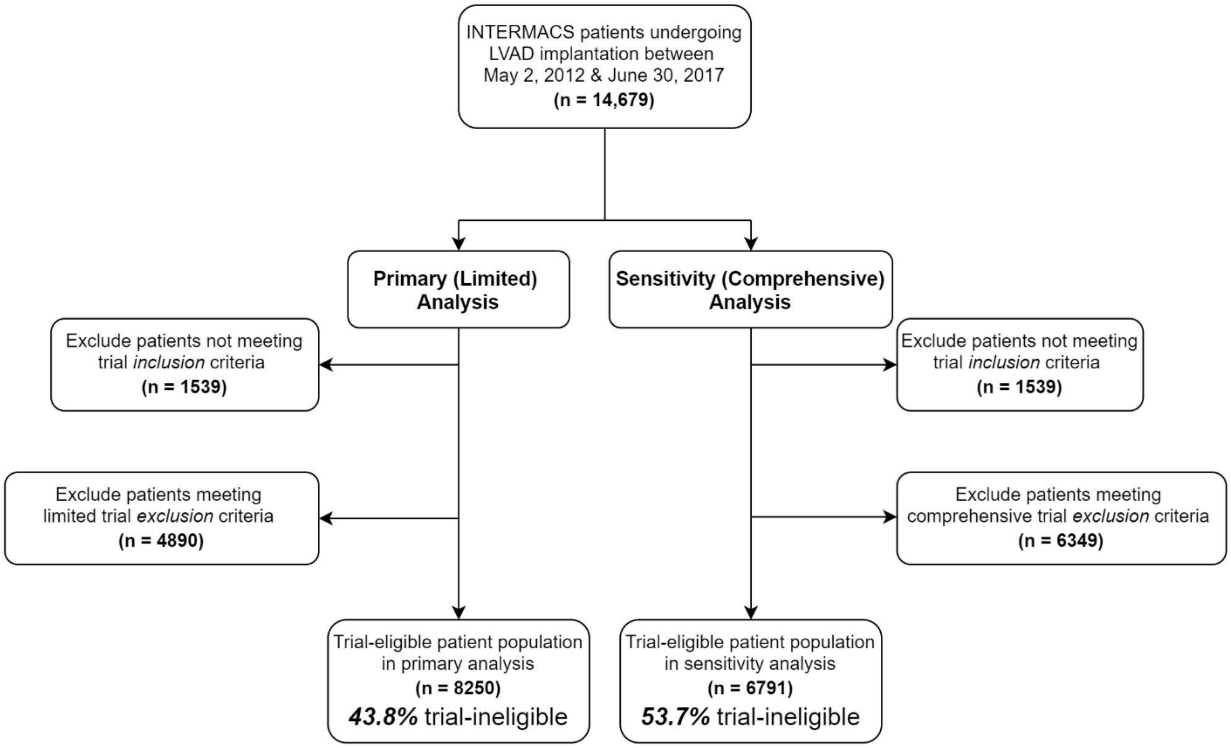

CONSORT diagram of primary (limited) and sensitivity (comprehensive) analyses identifying trial-eligible and trial-ineligible patients in the Interagency Registry for Mechanically Assisted Circulatory Support (INTERMACS). LVAD, left ventricular assist device.

© 2021 Brescia AA et al. *JAMA Network Open.*

eFigure 2. Ineligibility by Year

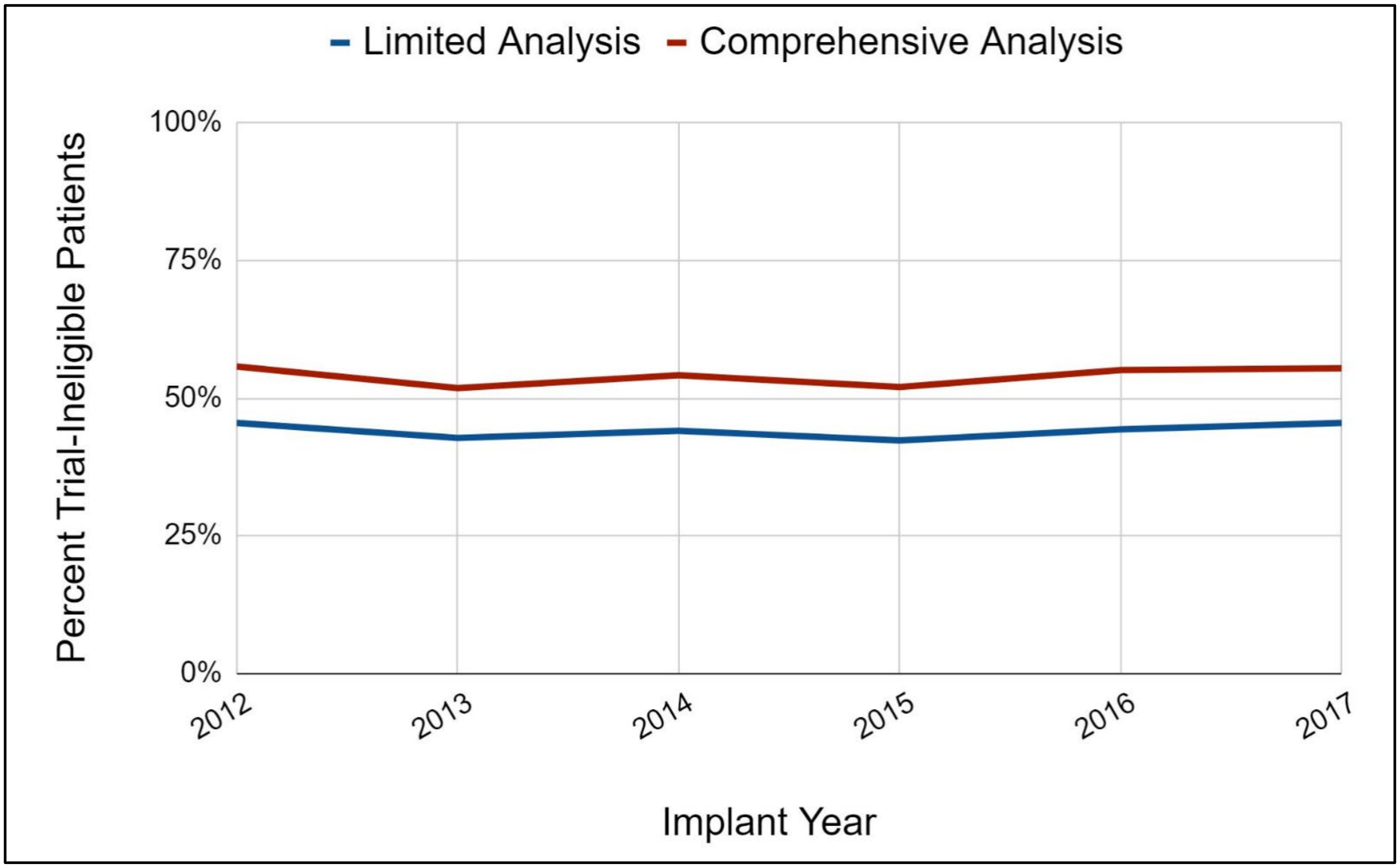

Annual trend in overall percent of trial-ineligible patients in the Interagency Registry for Mechanically Assisted Circulatory Support (INTERMACS) database for primary (limited, blue) and sensitivity (comprehensive, red) analyses.

© 2021 Brescia AA et al. *JAMA Network Open.*

eFigure 3. Distribution of Ineligibility Criteria

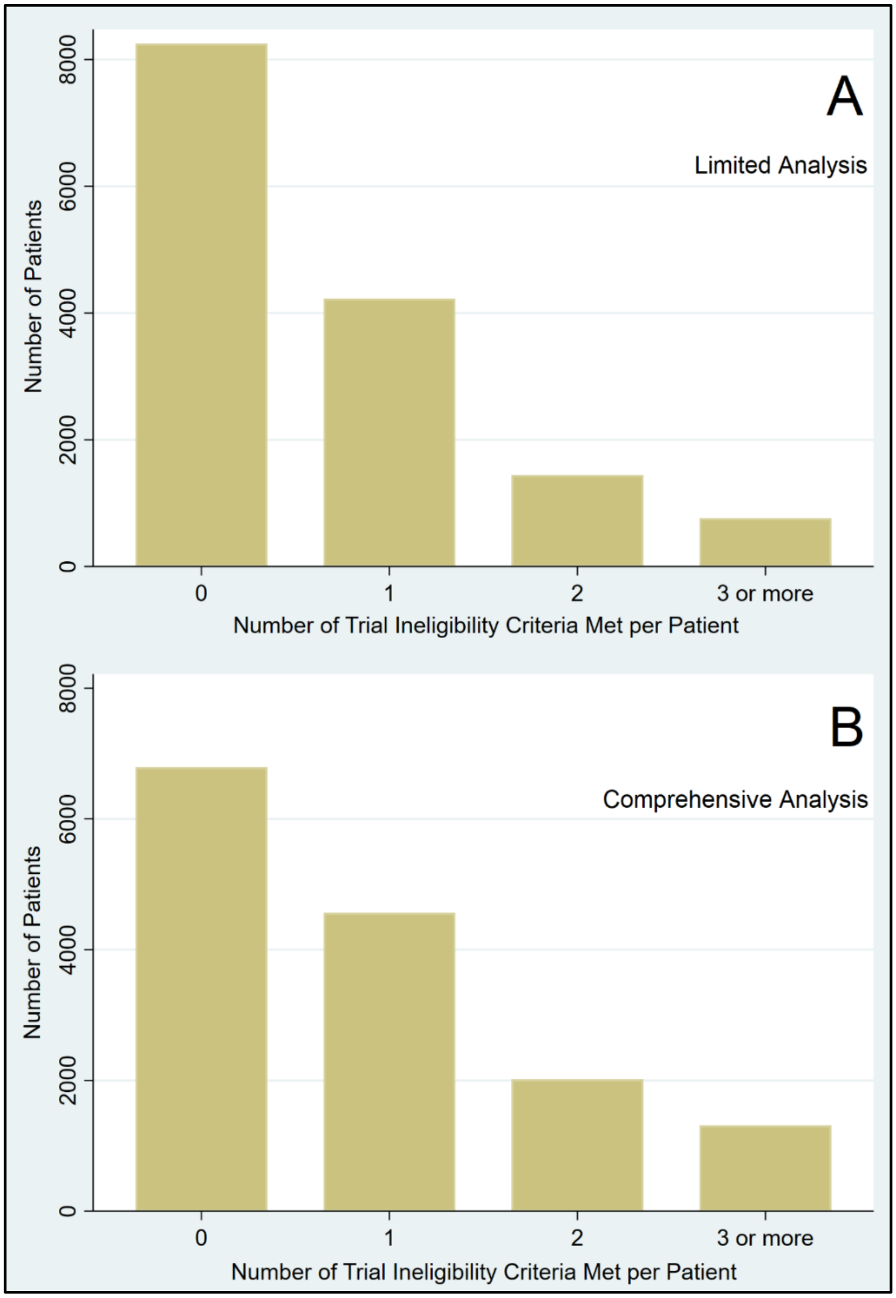

Number of ineligibility criteria met per patient for the **A)** primary analysis (limited criteria) and **B)** sensitivity analysis (comprehensive criteria). Patients meeting 0 ineligibility criteria are trial-eligible.

© 2021 Brescia AA et al. *JAMA Network Open.*

eFigure 4. Frequency of Ineligibility Criteria

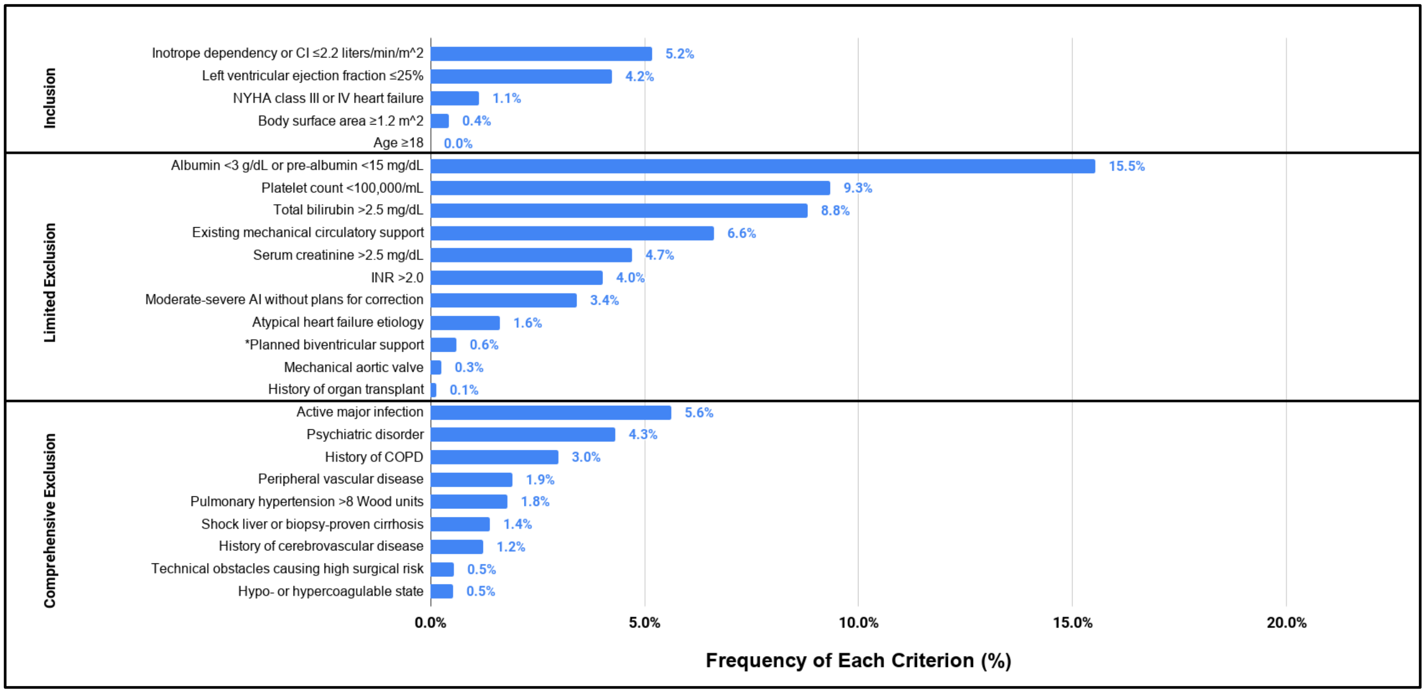

Frequency of trial-ineligible patients by inclusion criteria (top group), limited analysis exclusion criteria (middle group), and comprehensive analysis exclusion criteria (bottom group). *The frequency of exclusion due to planned biventricular support is shown in the Figure for the limited analysis definition; for the comprehensive analysis, n=716 (4.9%) of patients met this exclusion criterion (**eTable 1**). The bottom panel depicts the additional variables included in the comprehensive analysis, while the middle panel includes variables which are in both the limited and comprehensive analyses. CI, cardiac index; COPD, chronic obstructive pulmonary disorder; INR, International Normalized Ratio; NYHA, New York Heart Association.

© 2021 Brescia AA et al. *JAMA Network Open.*

eFigure 5. Cumulative Incidence of Mortality With Transplant as a Competing Risk

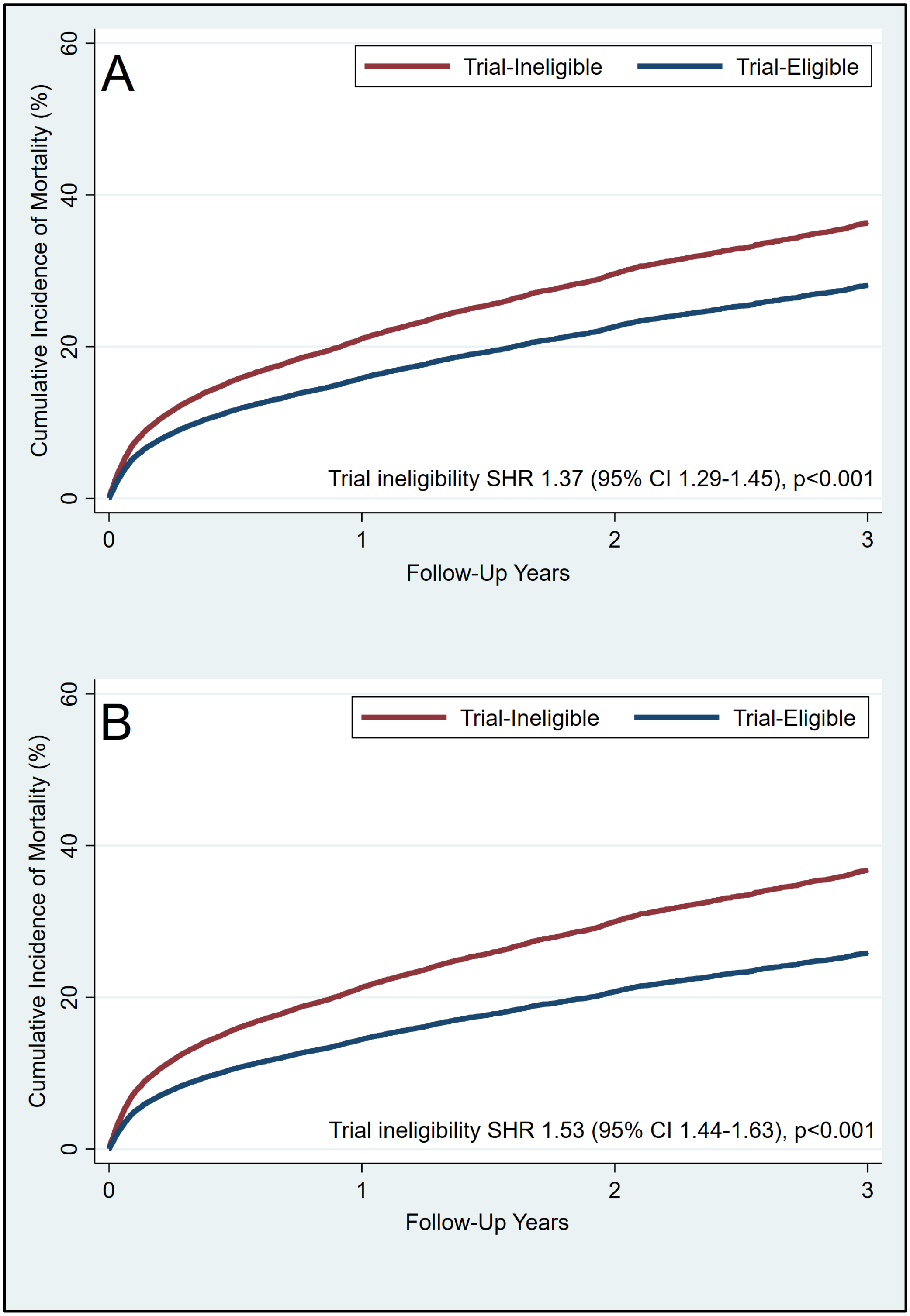

Cumulative incidence of mortality with cardiac transplantation as a competing risk by trial eligibility status for the **A)** primary analysis (limited criteria) and **B)** sensitivity analysis (comprehensive criteria). CI, confidence interval; SHR, subhazard ratio.

© 2021 Brescia AA et al. *JAMA Network Open.*

eFigure 6. Mortality by Eligibility Status Stratified by Device Type

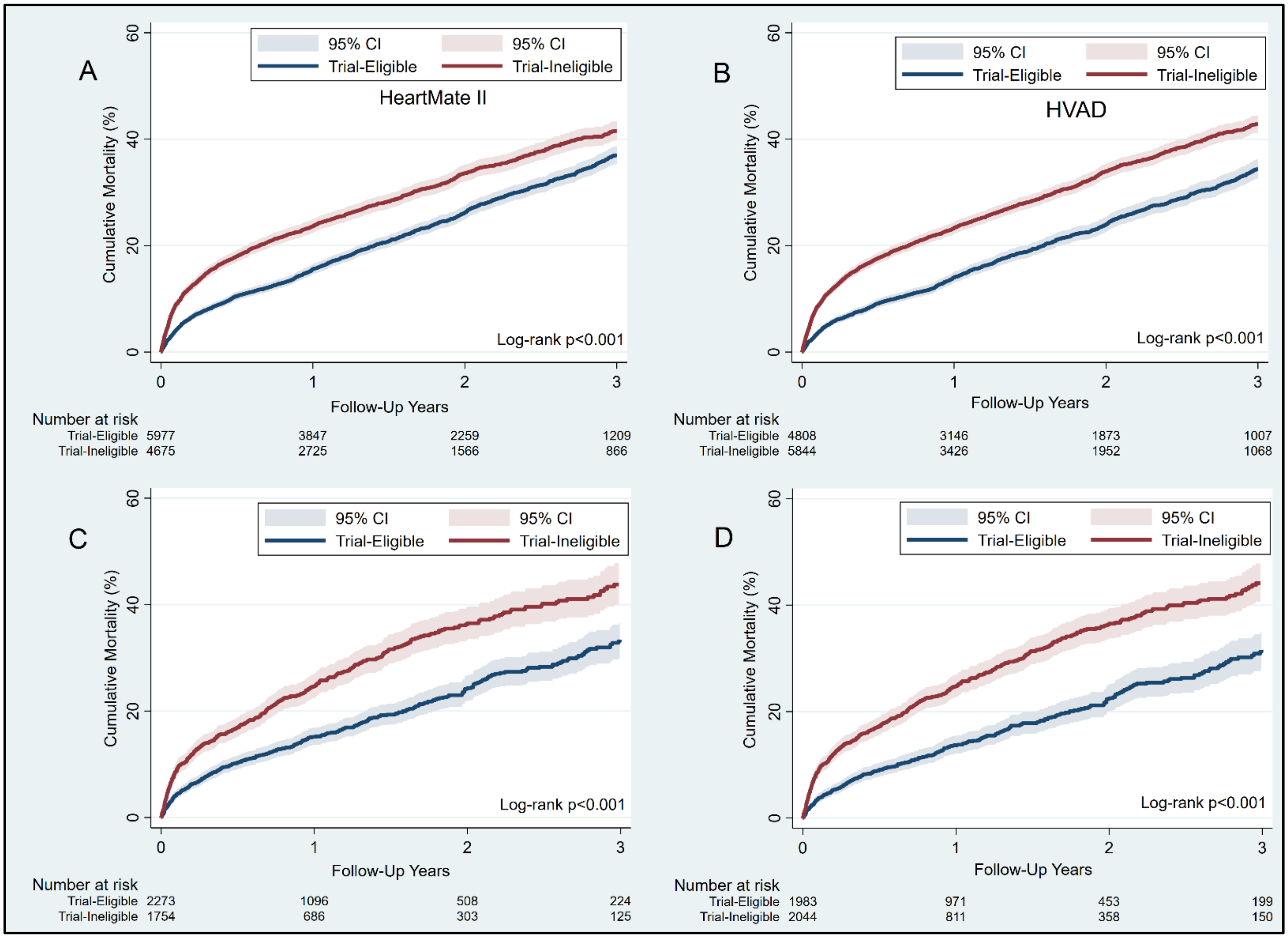

Kaplan-Meier estimated mortality by trial eligibility status and stratified by device type for the **A)** HeartMate II primary (limited) analysis, **B)** HVAD limited analysis, **C)** HeartMate II sensitivity (comprehensive) analysis, and **D)** HVAD comprehensive analysis. CI, confidence interval.

© 2021 Brescia AA et al. *JAMA Network Open.*

eFigure 7. Mortality by INTERMACS Profile

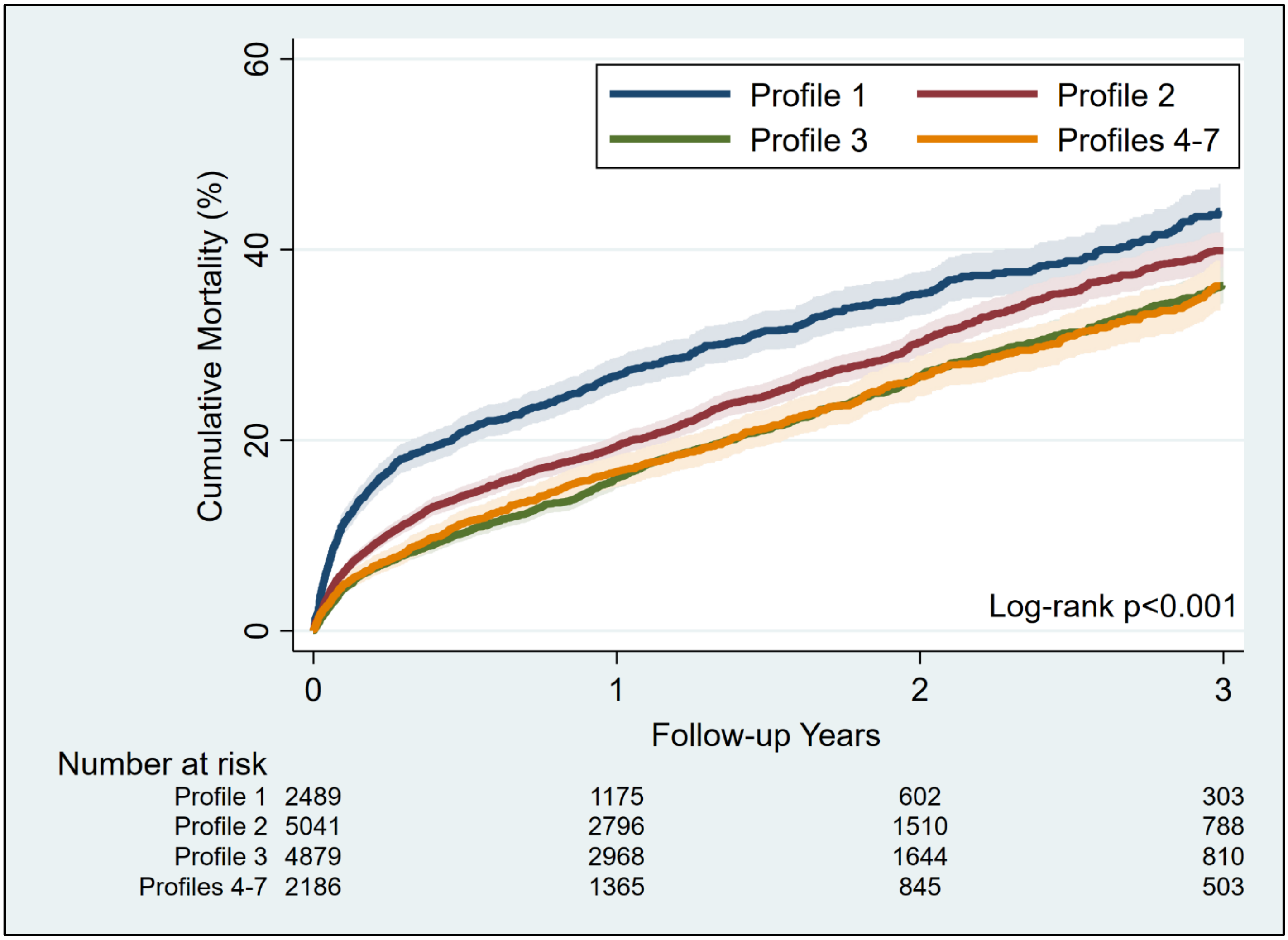

Kaplan-Meier estimated mortality by Interagency Registry for Mechanically Assisted Circulatory Support (INTERMACS) patient Profile.

© 2021 Brescia AA et al. *JAMA Network Open.*

eFigure 8. Mortality by Eligibility Stratified by INTERMACS Profile for Limited Criteria

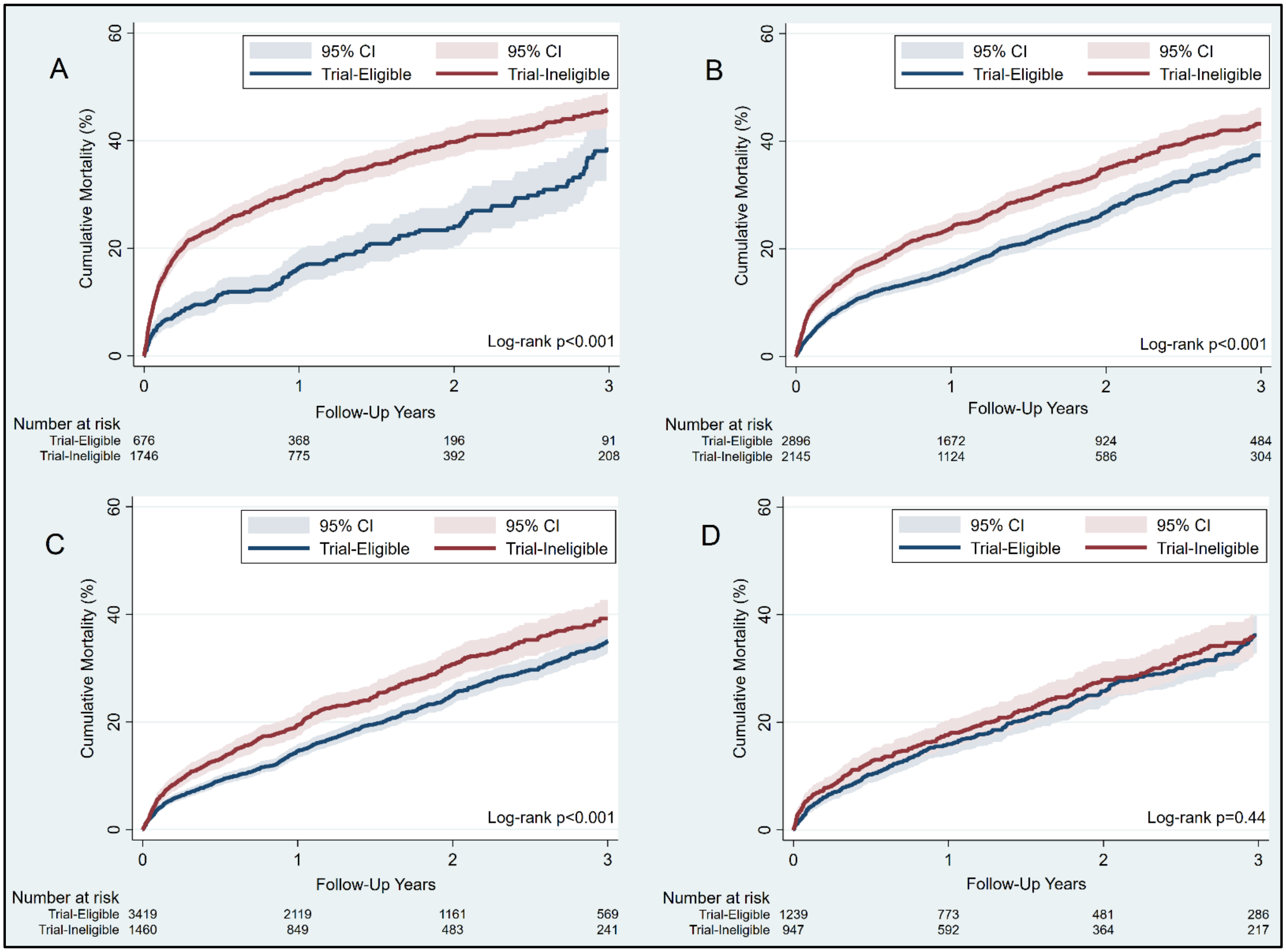

Kaplan-Meier estimated mortality by trial eligibility and stratified by Interagency Registry for Mechanically Assisted Circulatory Support (INTERMACS) patient Profiles for the primary analysis (limited criteria). **A)** Profile 1 **B)** Profile 2 **C)** Profile 3 **D)** Profiles 4-7. CI, confidence interval.

© 2021 Brescia AA et al. *JAMA Network Open.*

eFigure 9. Mortality by Eligibility Stratified by INTERMACS Profile for Comprehensive Criteria

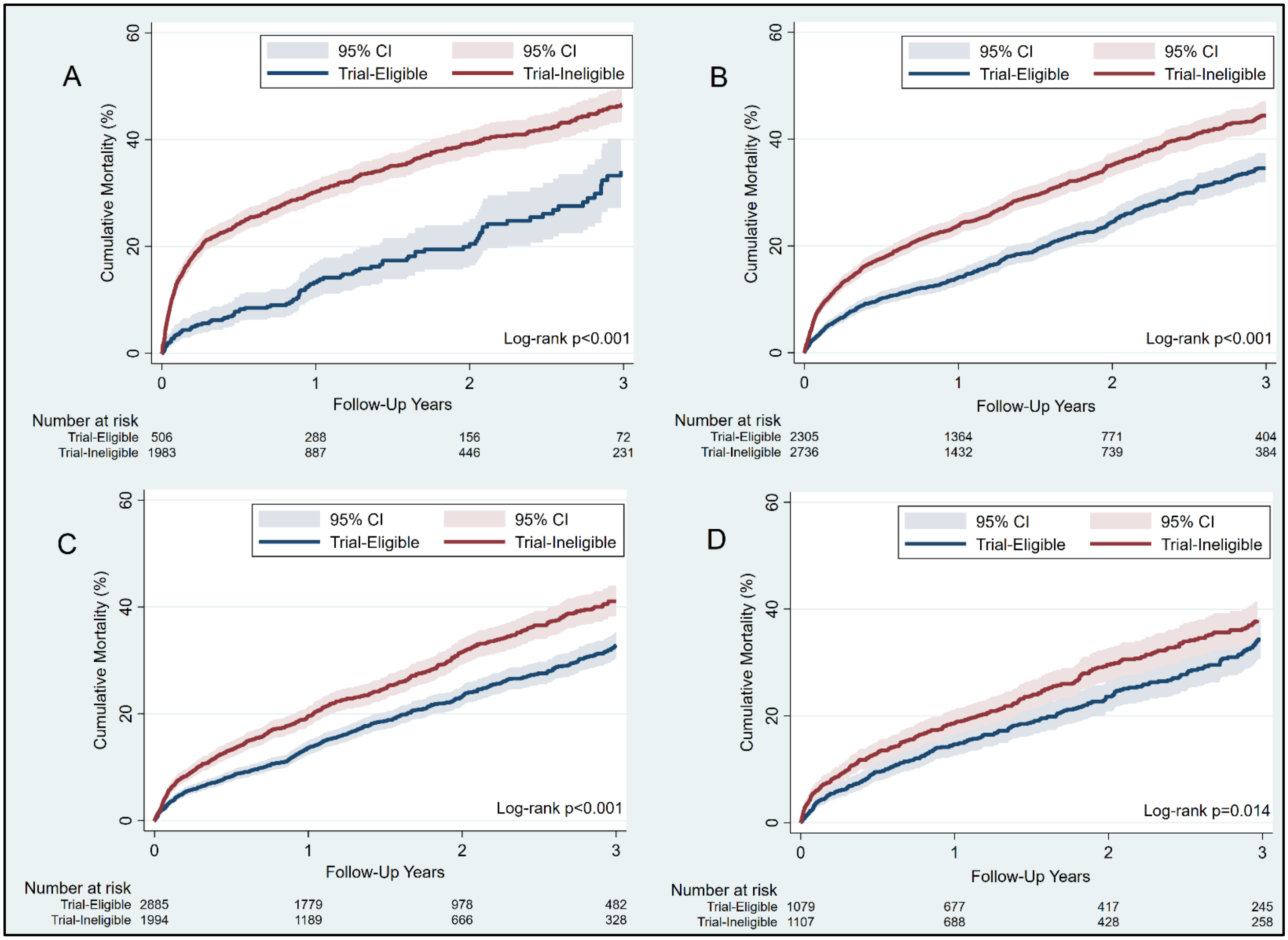

Kaplan-Meier estimated mortality by trial eligibility and stratified by Interagency Registry for Mechanically Assisted Circulatory Support (INTERMACS) patient Profiles for the sensitivity analysis (comprehensive criteria). **A)** Profile 1 **B)** Profile 2 **C)** Profile 3 **D)** Profiles 4-7. CI, confidence interval.

© 2021 Brescia AA et al. *JAMA Network Open.*
